# Supplementary material for: Design, Synthesis, and In Vivo and In Silico Evaluation of Coumarin Derivatives with Potential Antidepressant Effects
Source: Molecules. 2021 Sep 13;26(18):5556. doi: 10.3390/molecules26185556 (PMC8467986; doi:10.3390/molecules26185556)
Supplement: Supplementary file 1 [file molecules-26-05556-s001.zip › molecules-1360107-supplementary.pdf]

molecules

Supplementary materials

Design, Synthesis, and in Vivo and in Silico Evaluation of Coumarin Derivatives with Potential Antidepressant Effects

Xuekun Wang *, Hao Zhou, Xinyu Wang, Kang Lei and Shiben Wang *

School of Pharmaceutical Sciences, Liaocheng University, Liaocheng 252059, China; lcuyxyzhouhao@163.com (H.Z.); wangxinyuWXY1128@163.com (X.W.); leikang@lcu.edu.cn (K.L.)
* Correspondence: xuekunwang0610@126.com (X.W.); wangshiben110@163.com (S.W.); Tel.: +86-0635-823-9087

Pharmacology

1. **Forced swimming test (FST)** [1–3]

Male Kun-Ming mice (19–21 g) were used in the FSTs. On the test day, mice were assigned to different groups (n = 8 for each group). The synthesized compounds and the standard drug fluoxetine were administered as intraperitoneal injections. Control animals received a 3% aqueous solution of Tween 80. After 30 min, the mice were dropped one at a time into a Plexiglas cylinder (height 25 cm, diameter 10 cm, containing water to a height of 10 cm at 23–25 °C) and observed for 6 min. After the first 2 min of vigorous struggling, the animals were immobile. A mouse was judged immobile if it floated in the water in an upright position and made only slight movements to prevent sinking. The total duration of immobility was recorded during the last 4 min of the 6-min test.

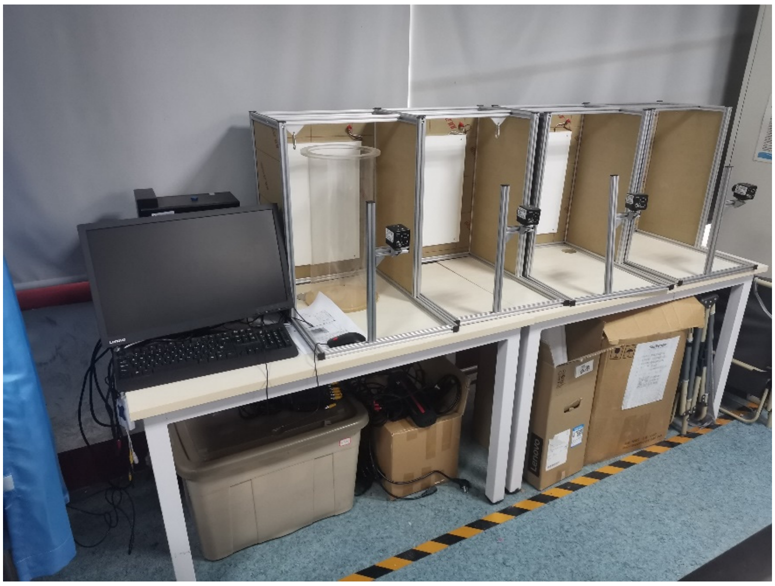

Figure S1. FST experimental instruments and equipment.

2. Tail suspension test (TST) [4,5]

The total duration of immobility induced by tail suspension was measured according to the method of Steru et al. (1985). Mice both acoustically and visually isolated were suspended 50 cm above the floor by adhesive tape placed approximately 1 cm from the tip of the tail. Immobility time was recorded during a 6-min test.

3. **Open-field test** [6,7]

Open-field tests were used to evaluate the exploratory activity of the animal. The investigated compound (5t suspended in aqueous Tween 80) was administered 60 min before the experiment. The study was carried out on mice according to Archer's method, with slight modifications. Each mouse was placed individually in the center of the open-field apparatus, and the locomotor activity was assessed. The open-field apparatus was a non-transparent plastic container (80 cm × 60 cm × 30 cm), with the underside divided into 48 units of size 10 cm*10 cm, without walls. The animals were gently placed in the center of the platform and were allowed to explore their surroundings. Hand-operated counters were used to score locomotion (ambulation, numbers of crossing lines with all four paws) and rearing frequencies (number of times an animal stood on its hind legs) for 3 min. The researchers, who did not know which groups had been treated, scored the behaviors in the open field. The experiments were performed in a dark room, and the apparatus was illuminated by a 60-W bulb giving a yellowish light, positioned 1 m above the center of the apparatus.

4. Build homology models methods using Discovery Studio (DS) 2020

(1) Load one or more known protein structures into a Molecule Window.

(2) Open an existing alignment of the template sequences and the model sequence into the Sequence Window. Note that the template sequences in the Sequence Window are automatically linked with the template structures in the Graphics View.

(3) Open the Macromolecules | Create Homology tools tools and click Build Homology Models... to open the Build Homology Models dialog

(4) Set the following parameters: eg: Input Sequence Alignment et.al.

(5) Click Run to start the calculation.

(6) Analyzing results: Analyzing output models, PDF energy and Troubleshooting

1. H-NMR, ^{13}C-NMR of compounds 4 and 5.

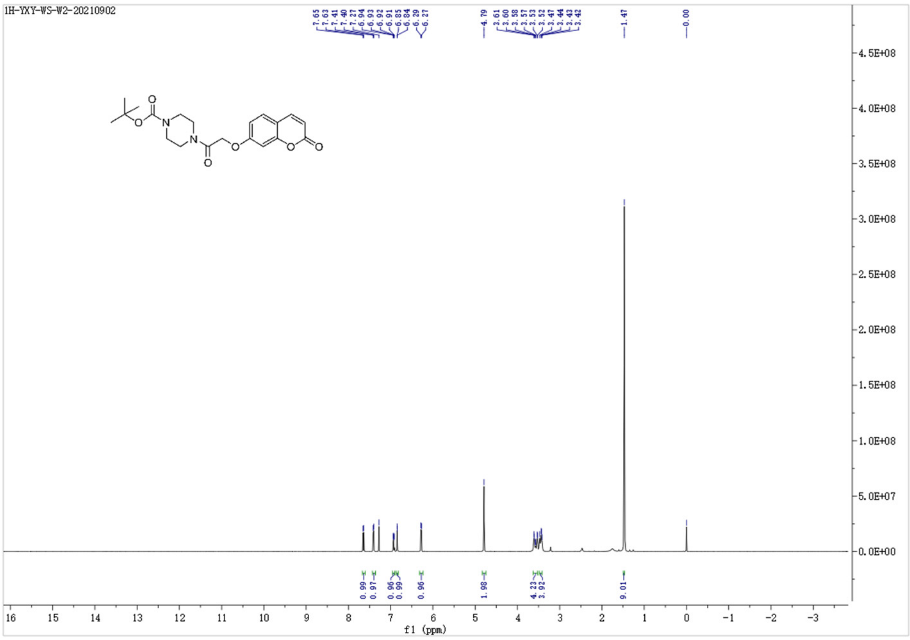

Figure S2. ^{1}H NMR spectrum **4.**

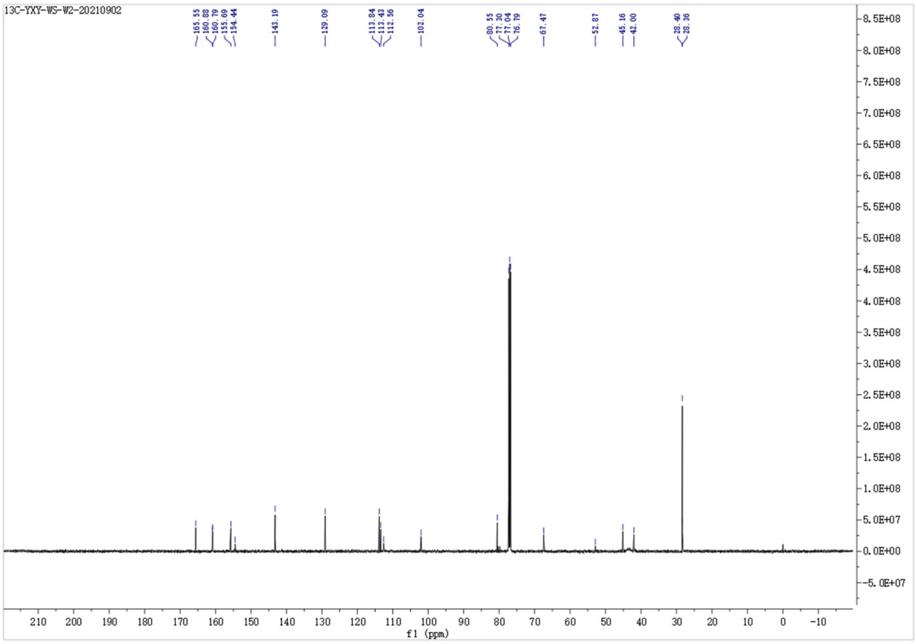

Figure S3. ^{13}C NMR spectrum **4.**

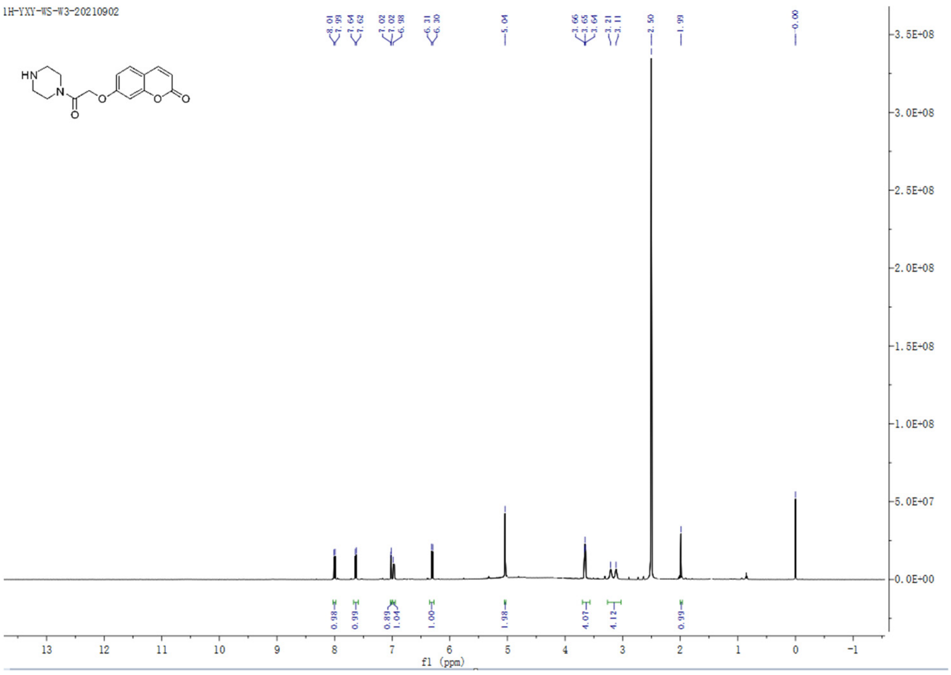

Figure S4. ^{1}H NMR spectrum **5.**

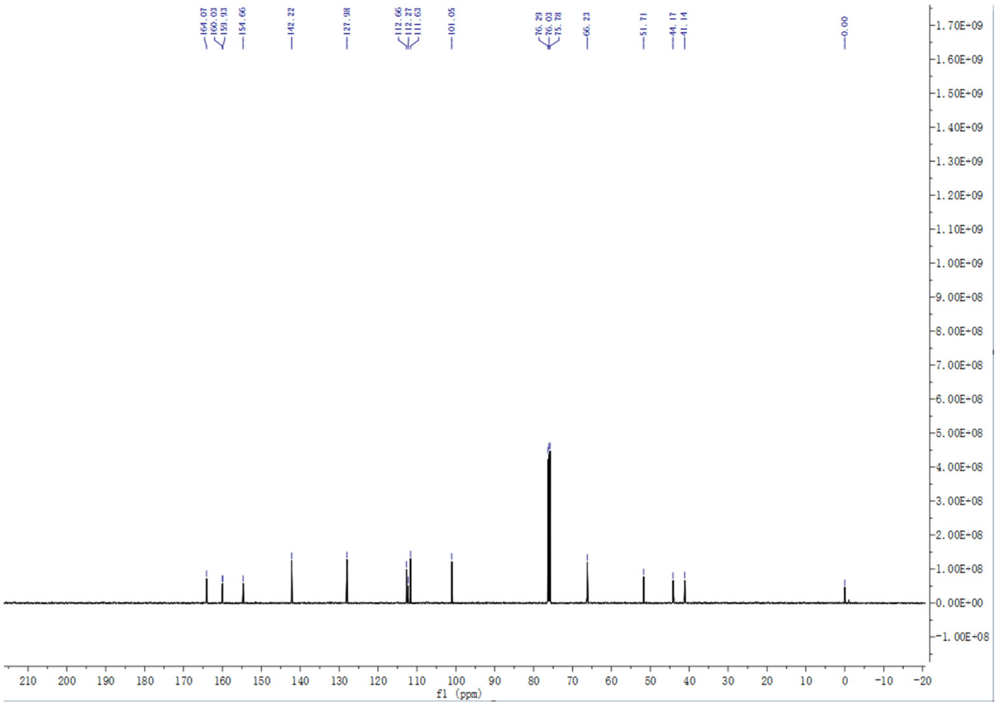

Figure S5. ^{13}C NMR spectrum **5.**

^{1}H-NMR, ^{13}C-NMR, and HRMS of target compounds

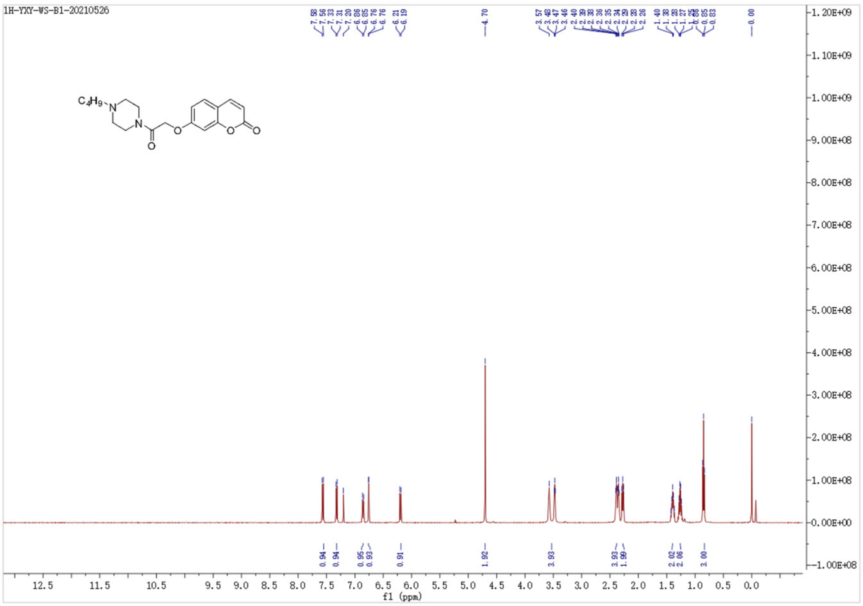

Figure S6. ^{1}H NMR spectrum **6a.**

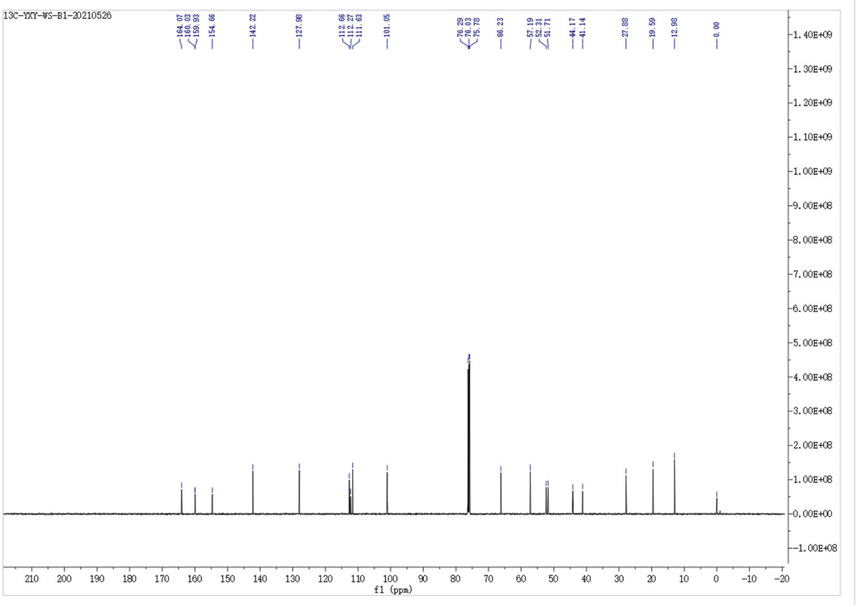

Figure S7. ^{13}C NMR spectrum **6a.**

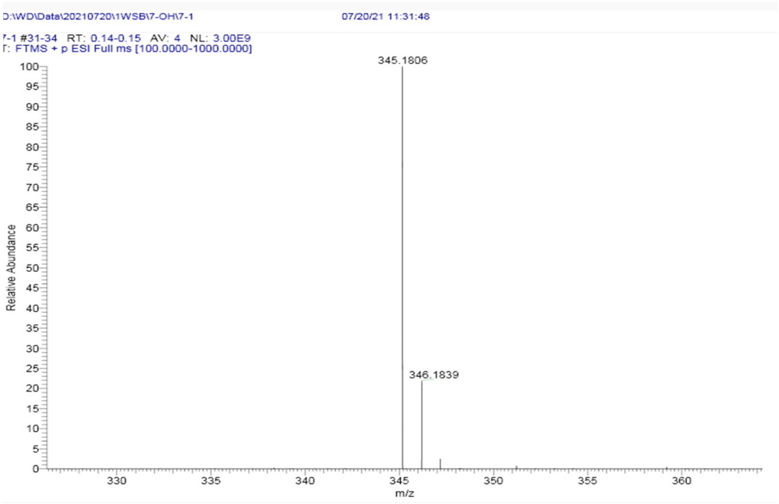

Figure S8. HRMS spectrum **6a.**

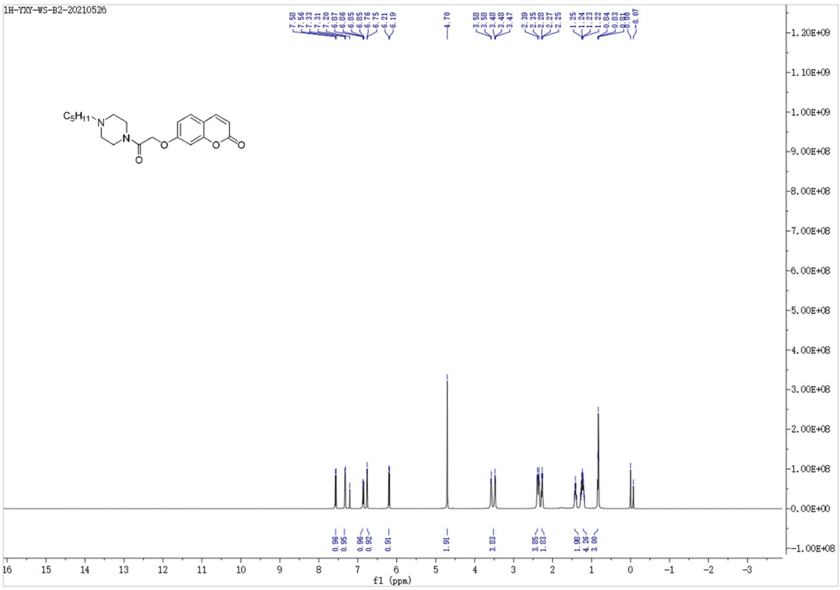

Figure S9. ^{1}H NMR spectrum **6b.**

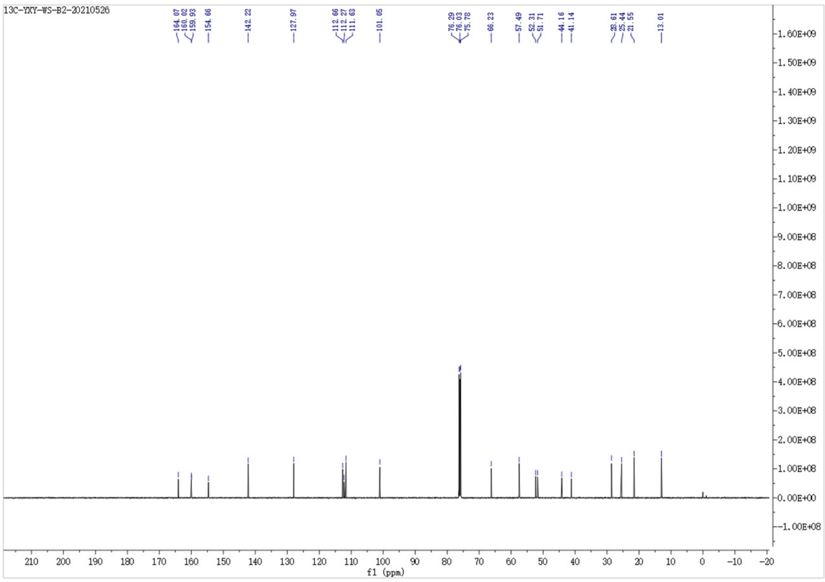

Figure S10. ^{13}C NMR spectrum **6b.**

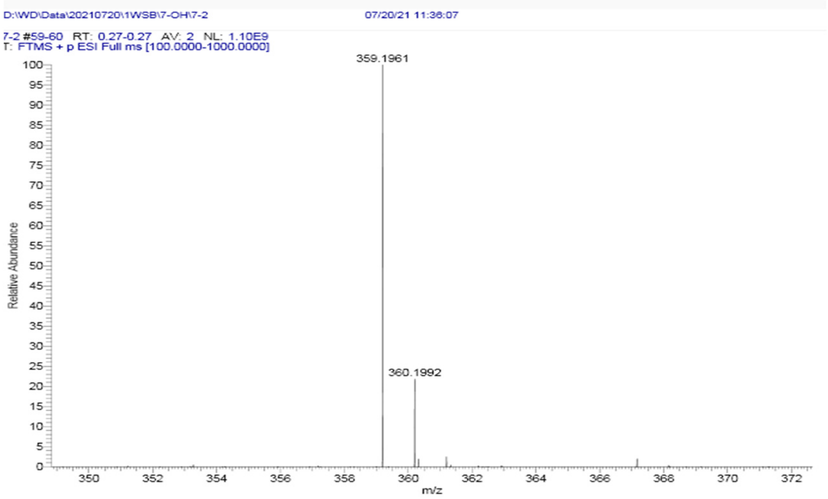

Figure S11. HRMS spectrum 6b.

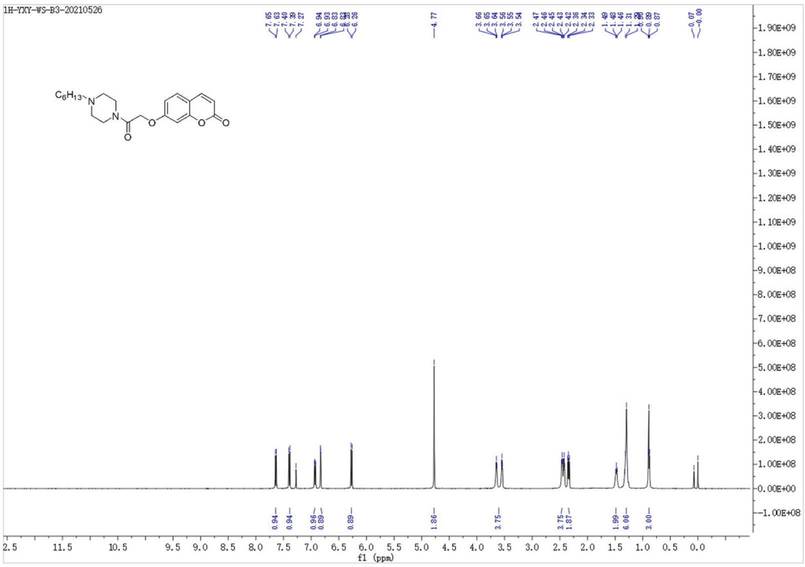

Figure S12. ^{1}H NMR spectrum **6c.**

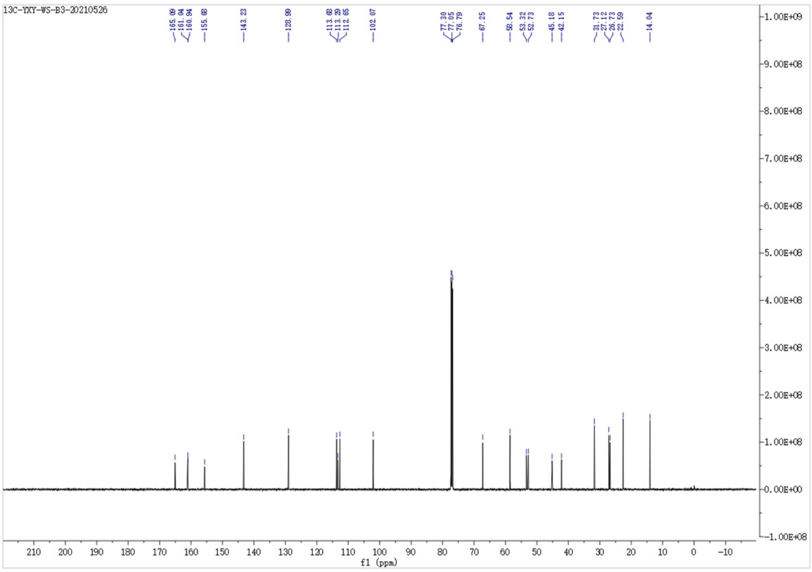

Figure S13. ^{13}C NMR spectrum **6c.**

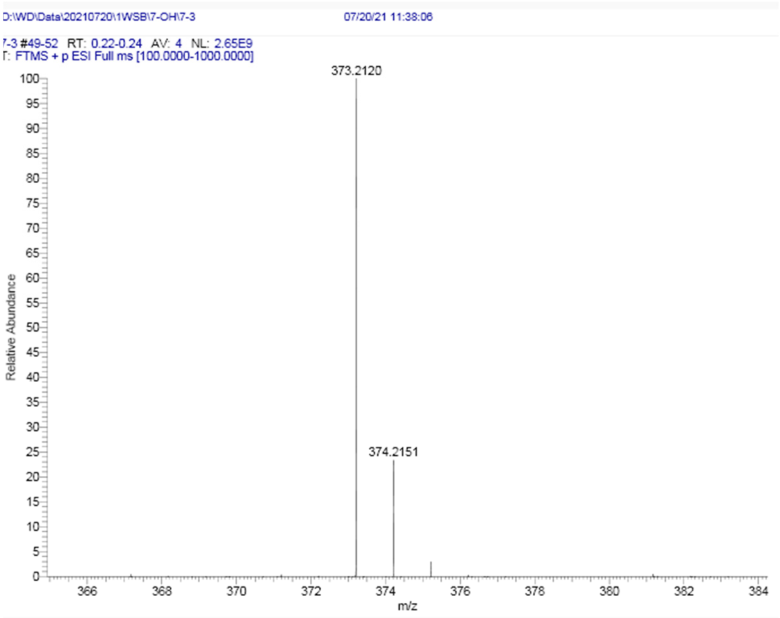

Figure S14. HRMS spectrum 6c.

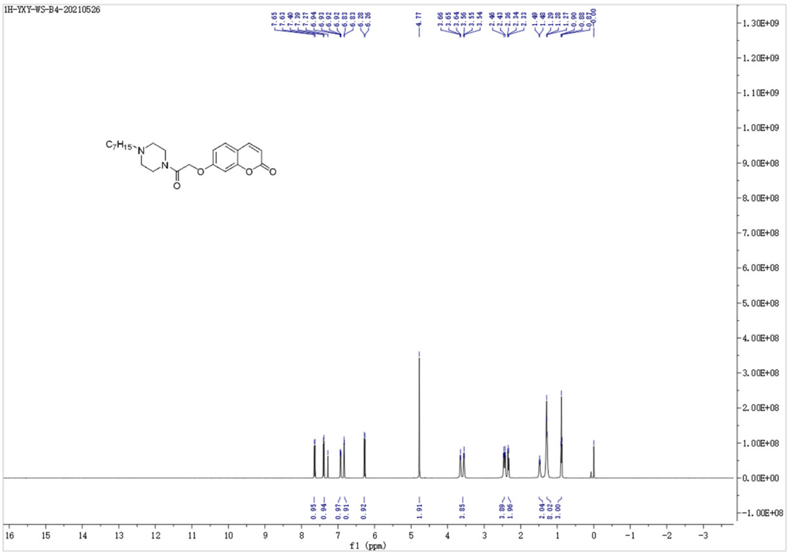

Figure S15. ^{1}H NMR spectrum **6d.**

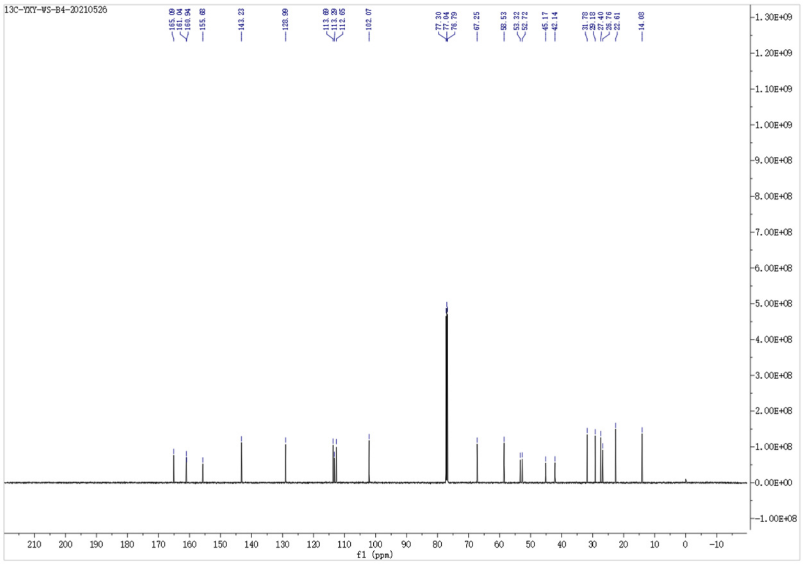

Figure S16. ^{13}C NMR spectrum **6d.**

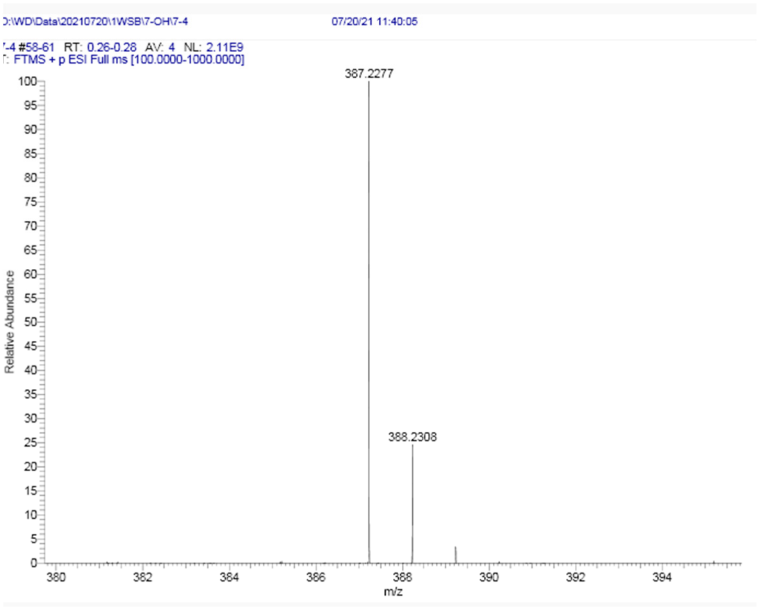

Figure S17. HRMS spectrum **6d.**

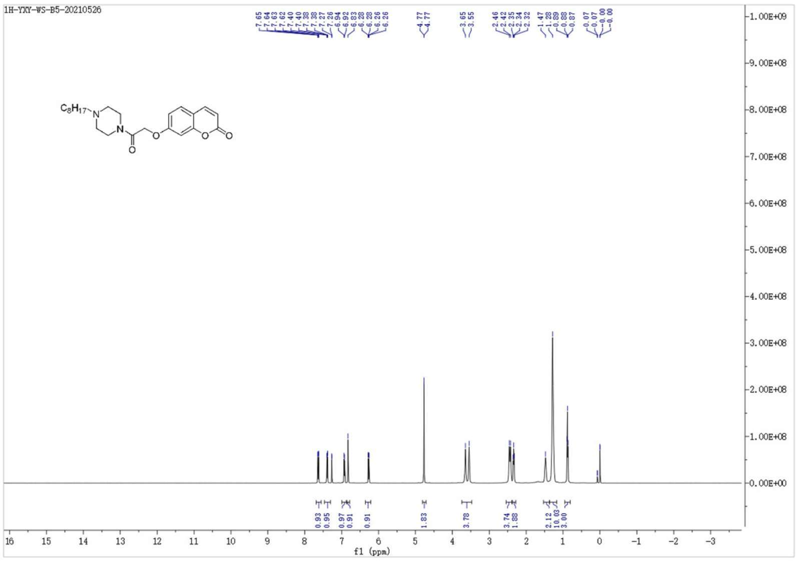

Figure S18. ^{1}H NMR spectrum **6e.**

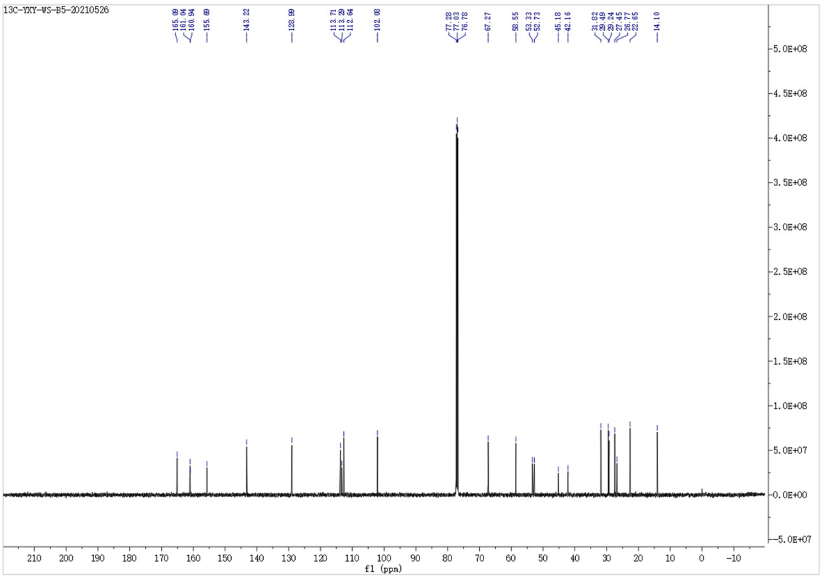

Figure S19. ^{13}C NMR spectrum **6e.**

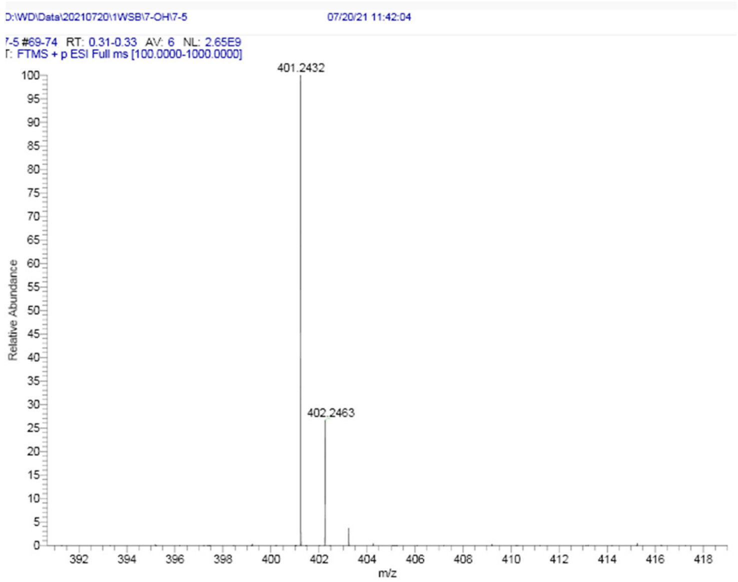

Figure S20. HRMS spectrum 6e.

Compound 6f

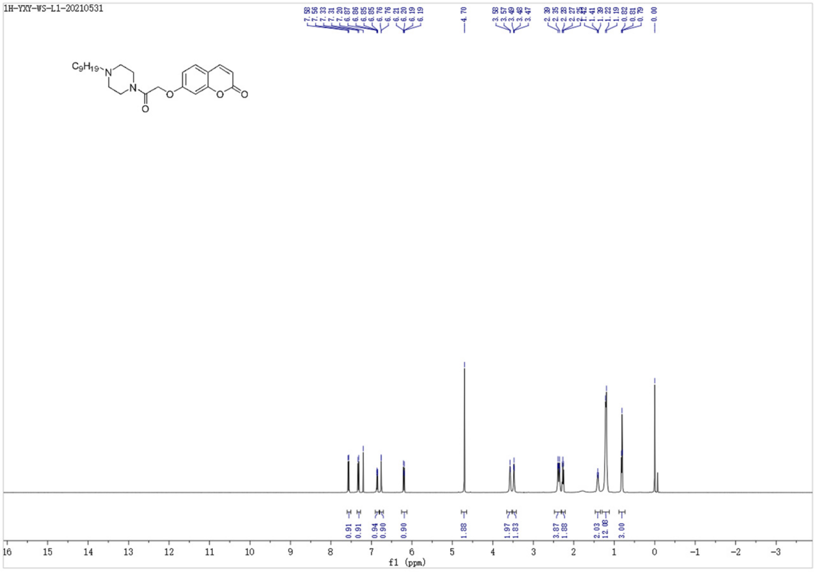

Figure S21. ^{1}H NMR spectrum **6f.**

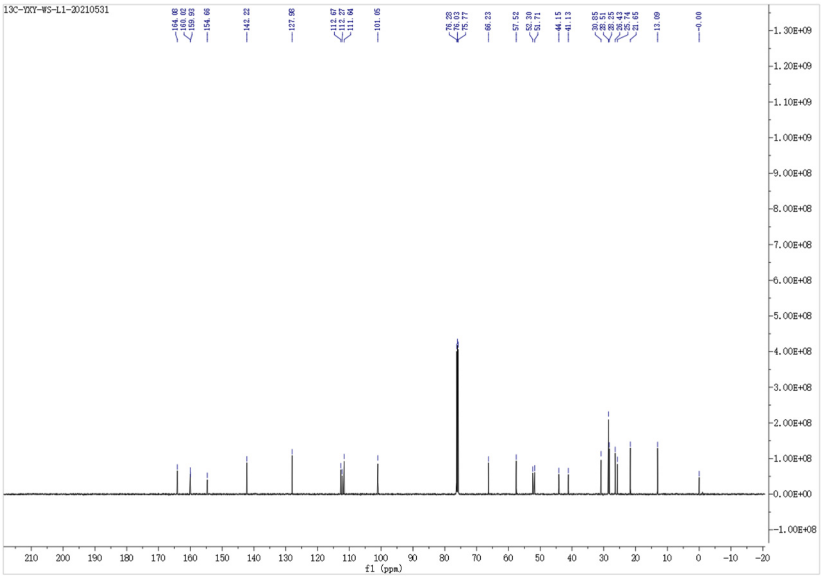

Figure S22. ^{13}C NMR spectrum **6f.**

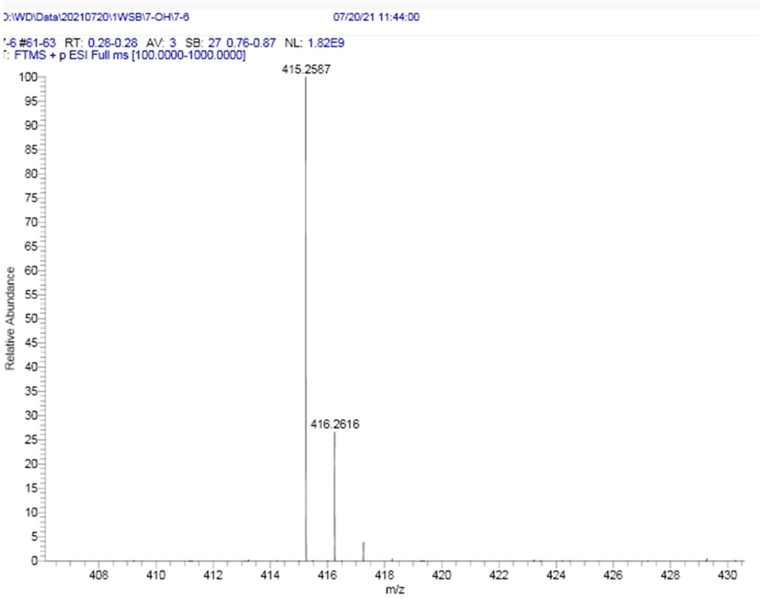

Figure S23. HRMS spectrum 6f.

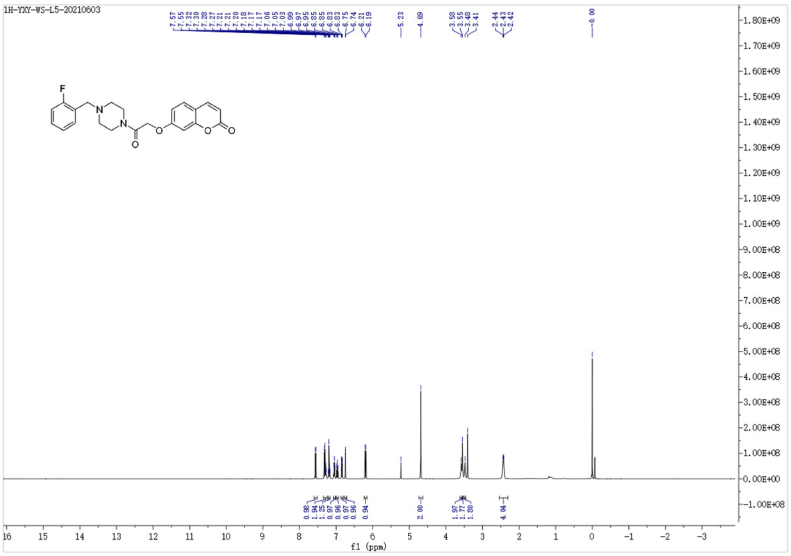

Figure S24. ^{1}H NMR spectrum **6g.**

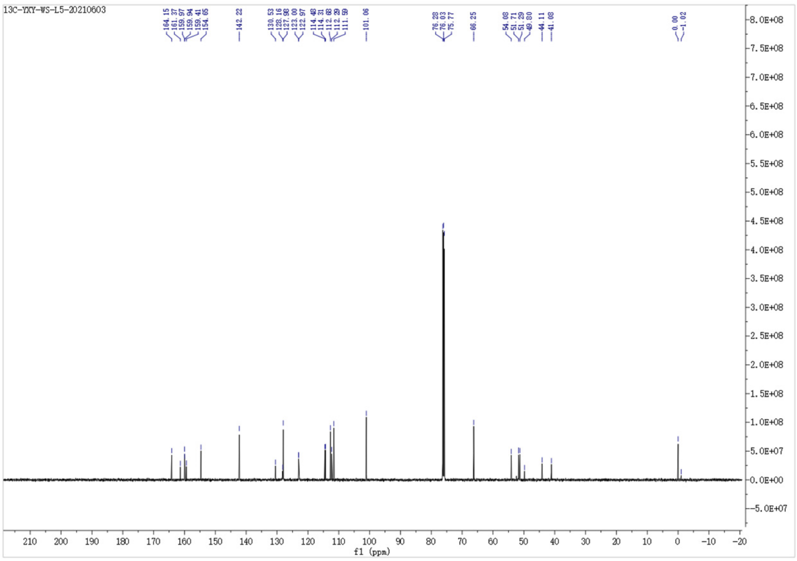

Figure S25. ^{13}C NMR spectrum **6g.**

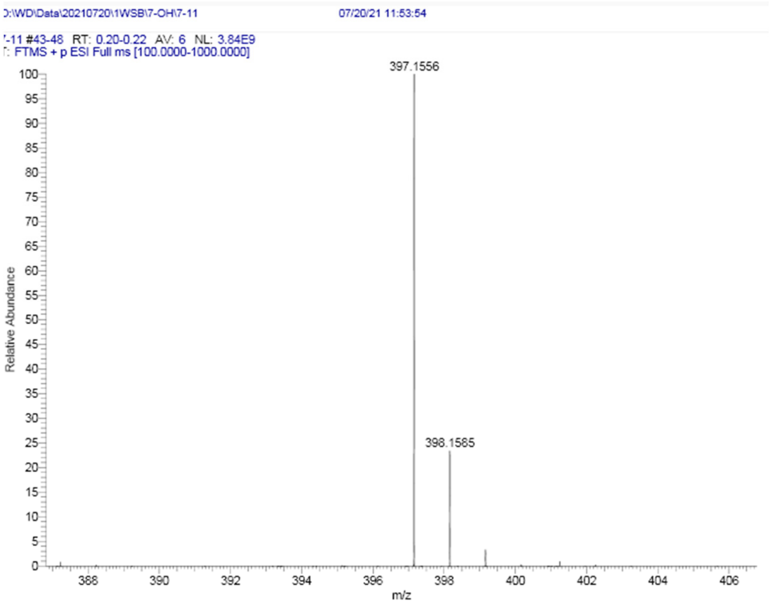

Figure S26. HRMS spectrum 6g.

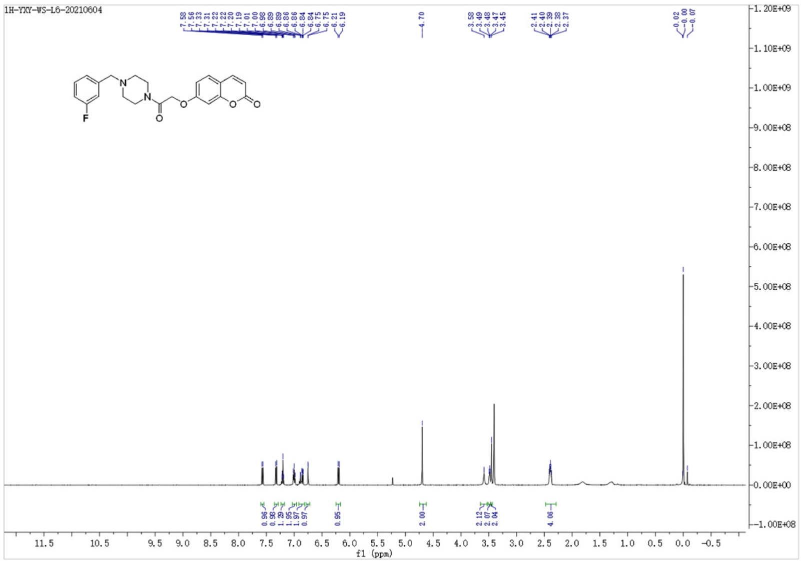

Figure S27. ^{1}H NMR spectrum **6h.**

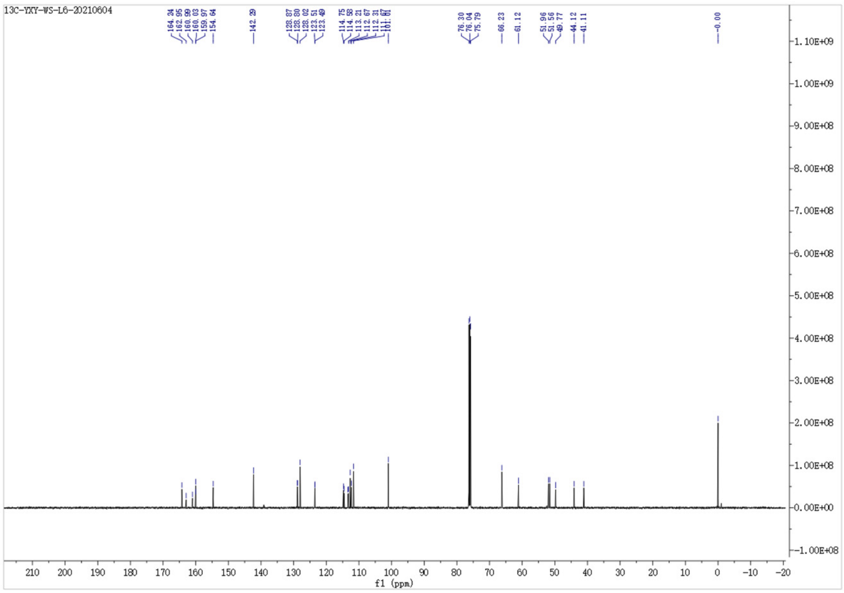

Figure S28. ^{13}C NMR spectrum **6h.**

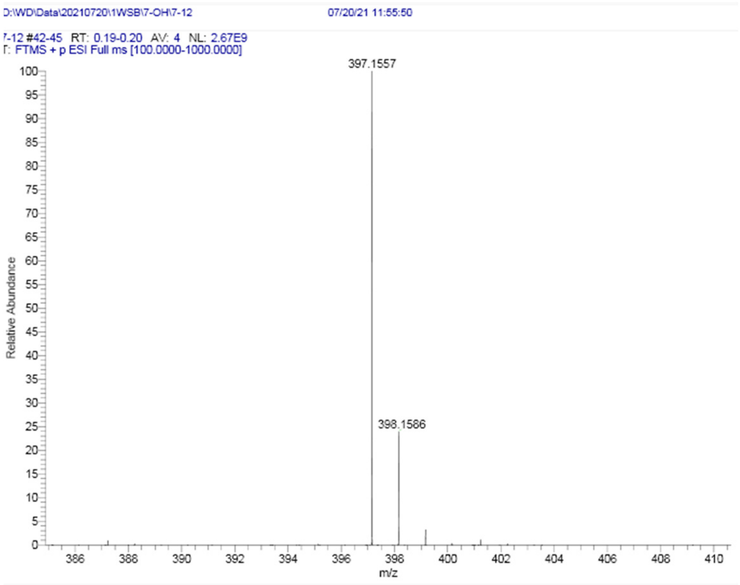

Figure S29. HRMS spectrum 6h.

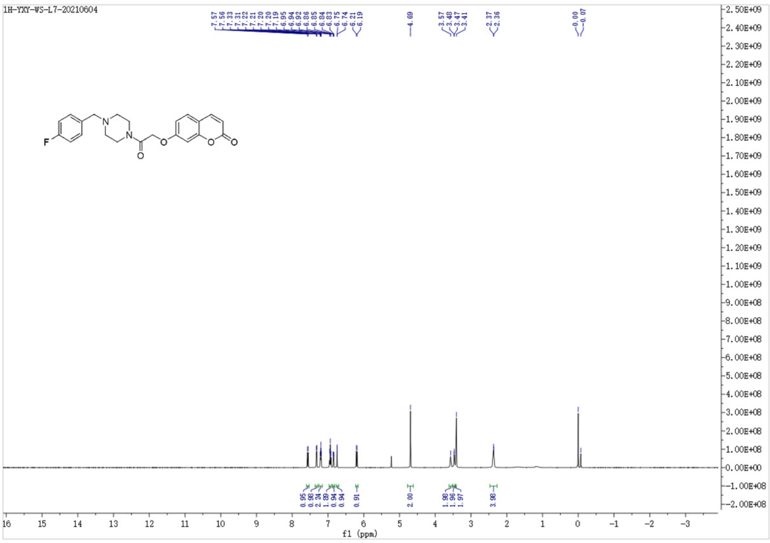

Figure S30. ^{1}H NMR spectrum **6i**.

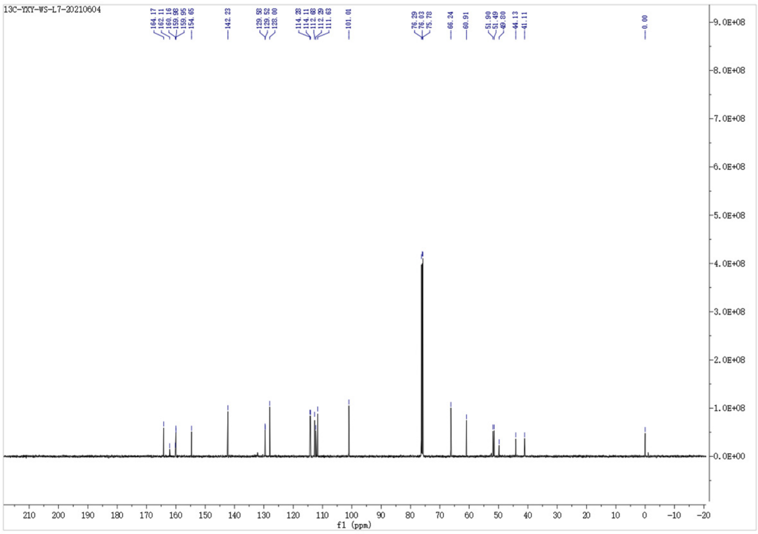

Figure S31. ^{13}C NMR spectrum **6i.**

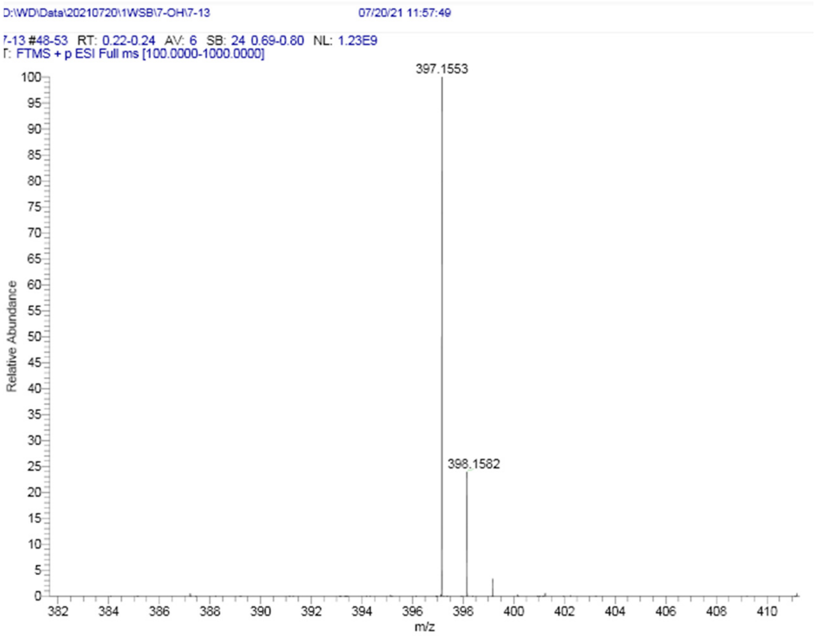

Figure S32. HRMS spectrum 6i.

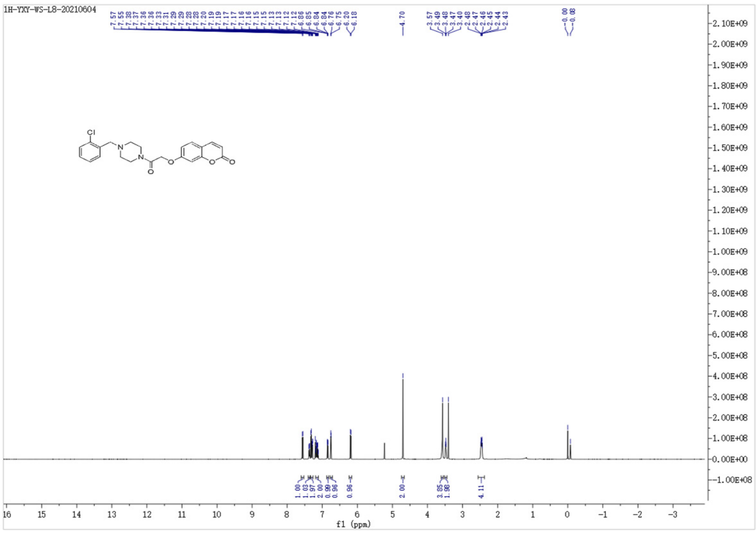

Figure S33. ^{1}H NMR spectrum **6j.**

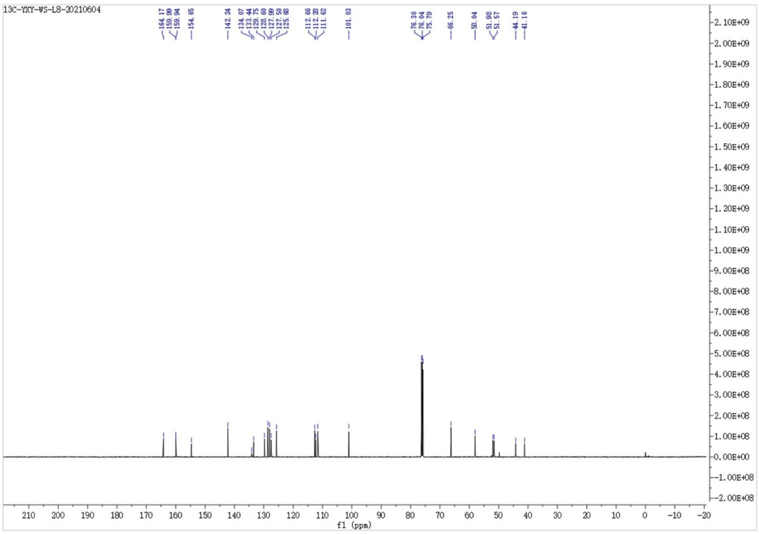

Figure S34. ^{13}C NMR spectrum **6j.**

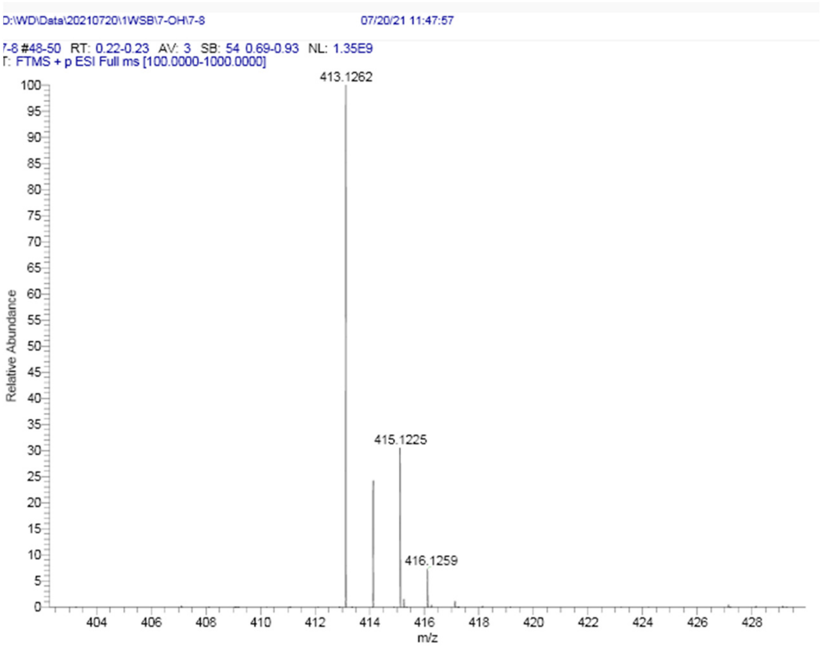

Figure S35. HRMS spectrum 6j.

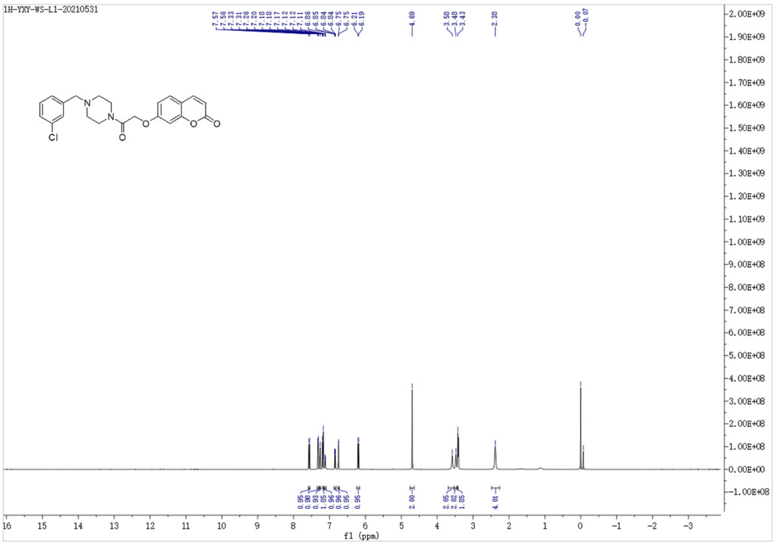

Figure S36. ^{1}H NMR spectrum **6k.**

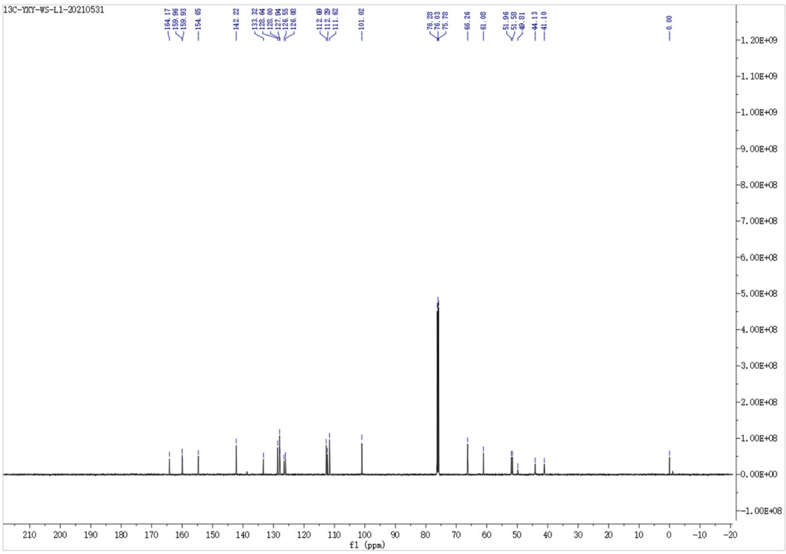

Figure S37. ^{13}C NMR spectrum **6k.**

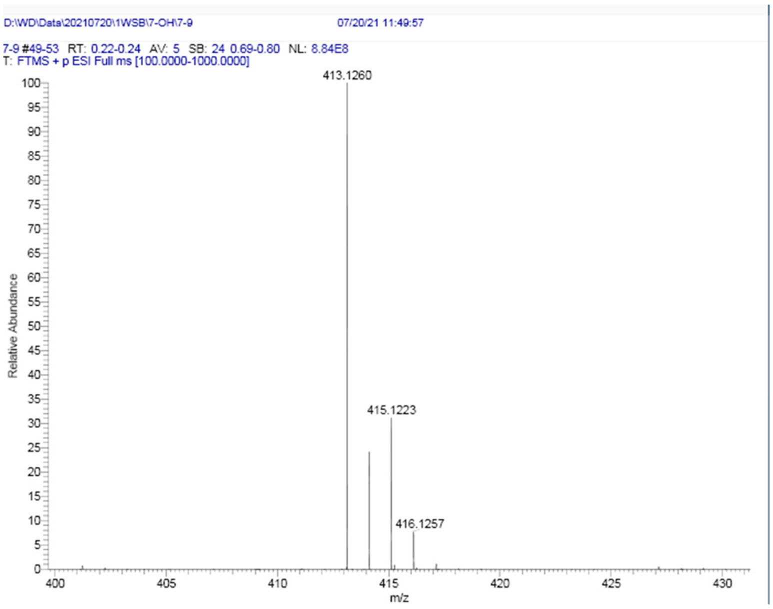

Figure S38. HRMS spectrum 6k.

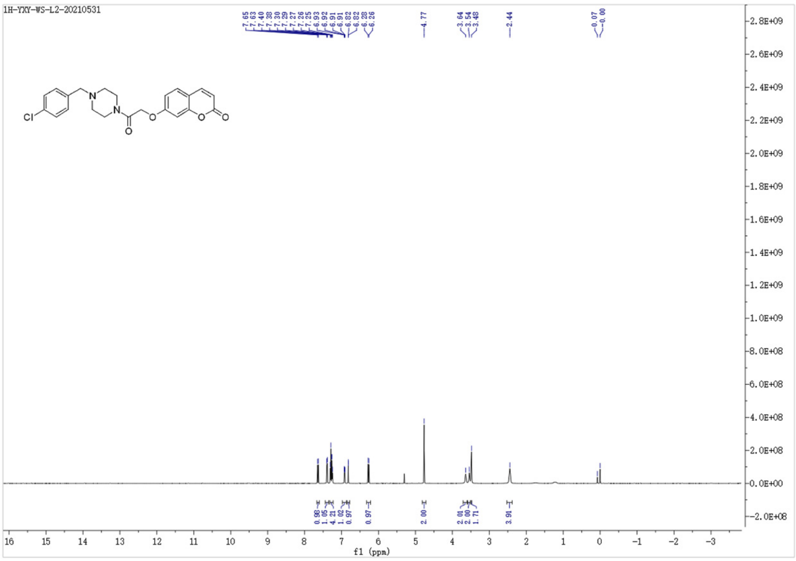

Figure S39. ^{1}H NMR spectrum **6l.**

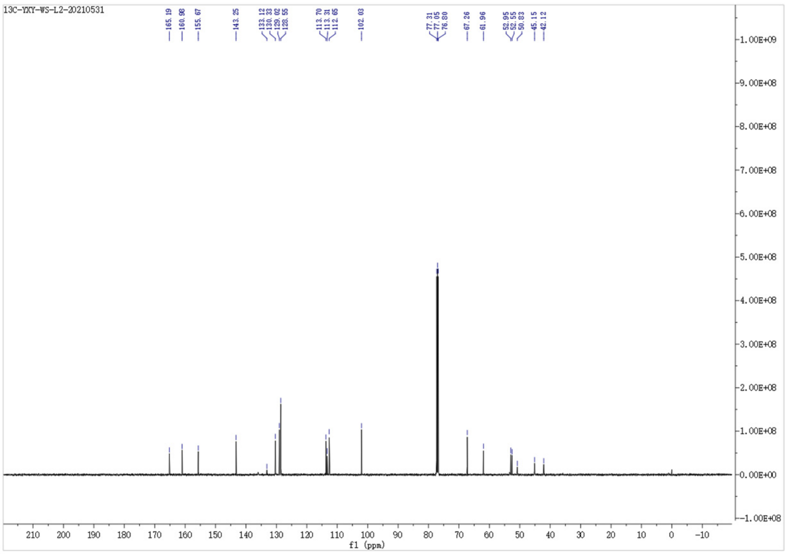

Figure S40. ^{13}C NMR spectrum **6l.**

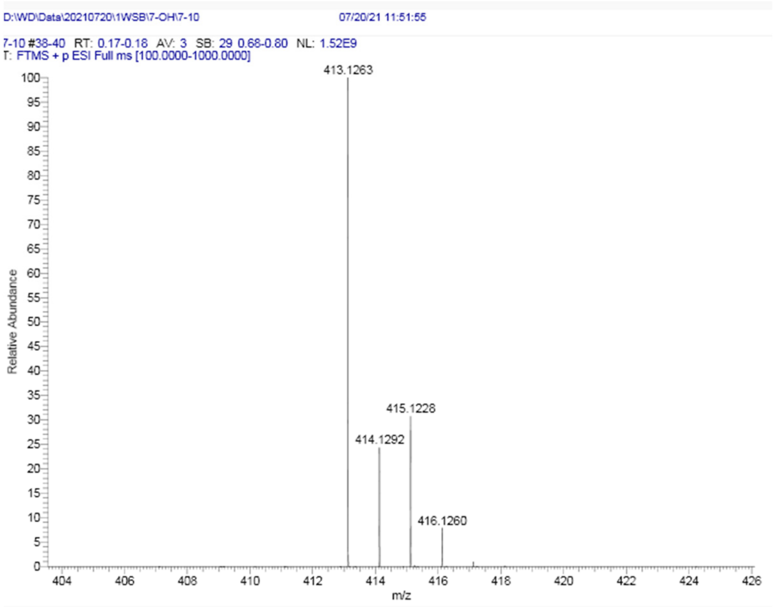

Figure S41. HRMS spectrum 6l.

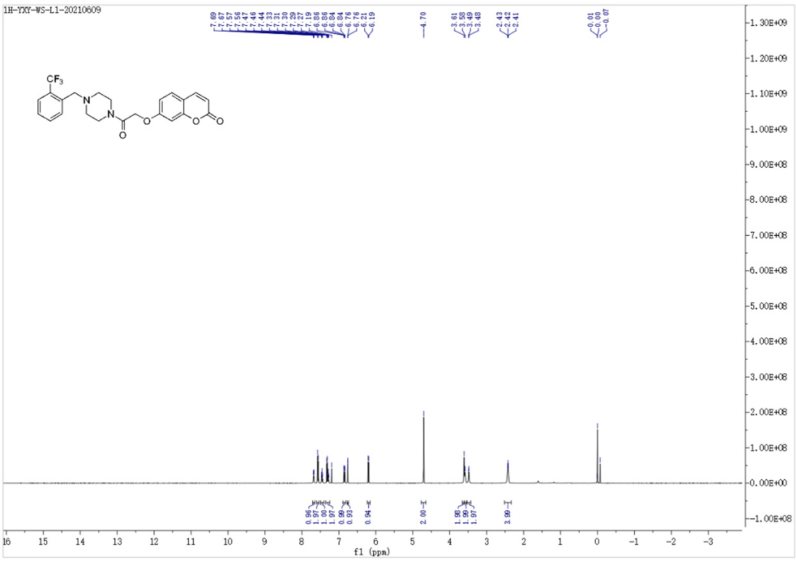

Figure S42. ^{1}H NMR spectrum **6m.**

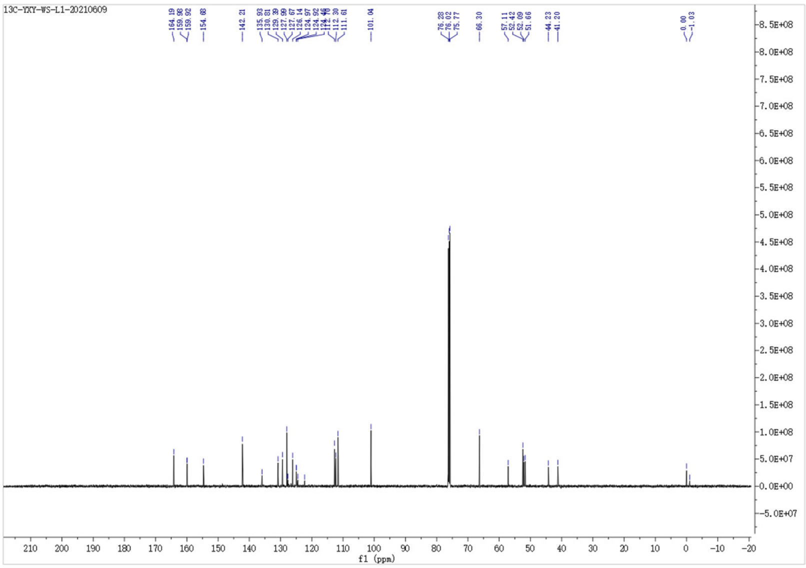

Figure S43. ^{13}C NMR spectrum **6m.**

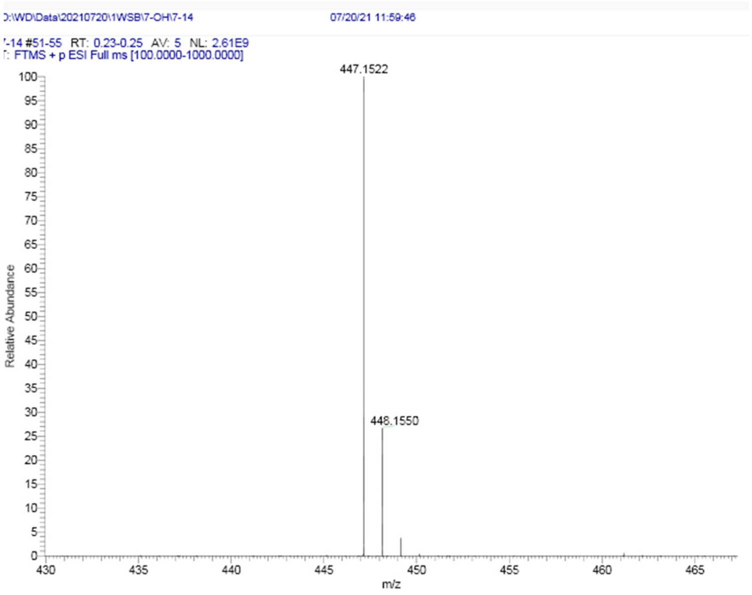

Figure S44. HRMS spectrum 6m.

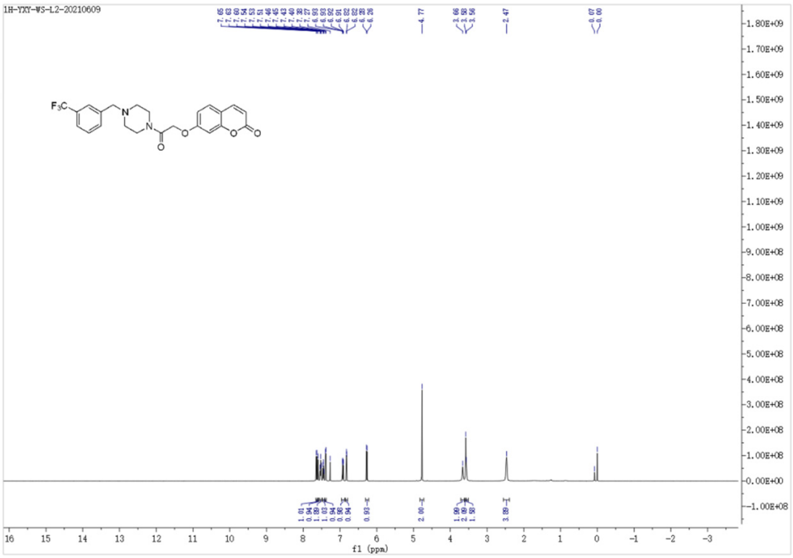

Figure S45. ^{1}H NMR spectrum **6n.**

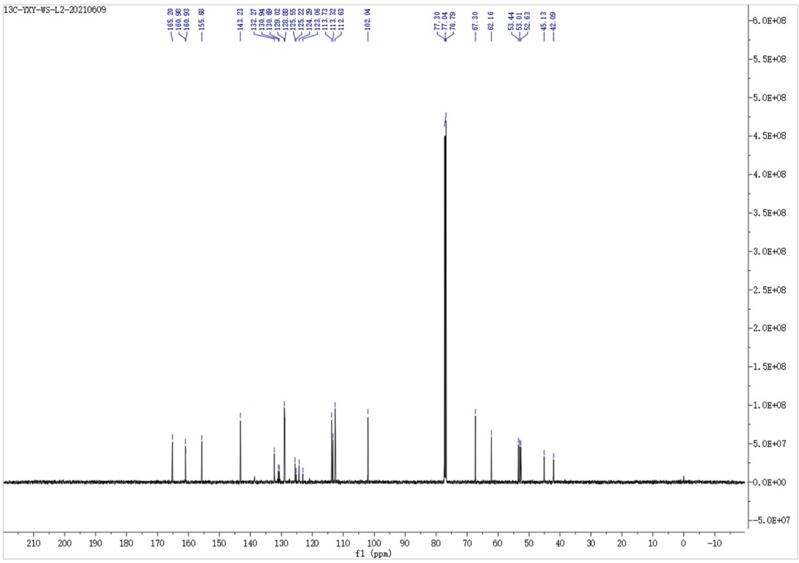

Figure S46. ^{13}C NMR spectrum **6n.**

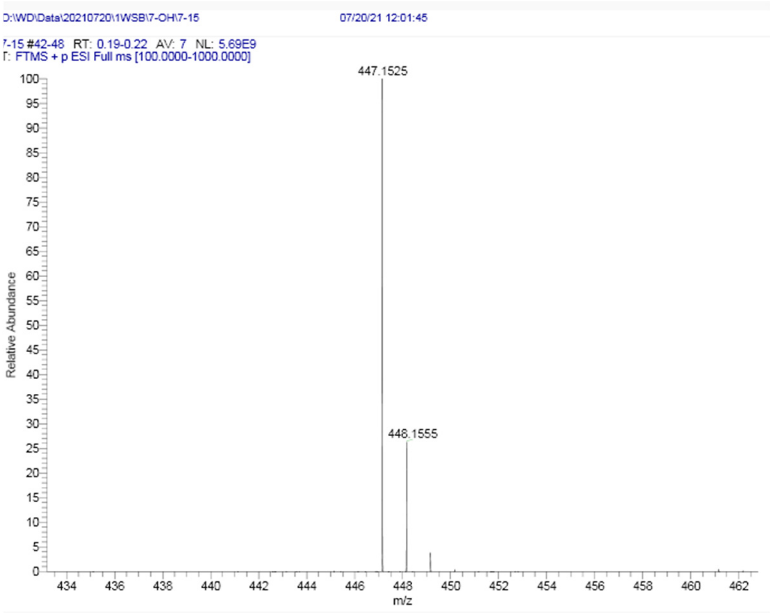

Figure S47. HRMS spectrum 6n.

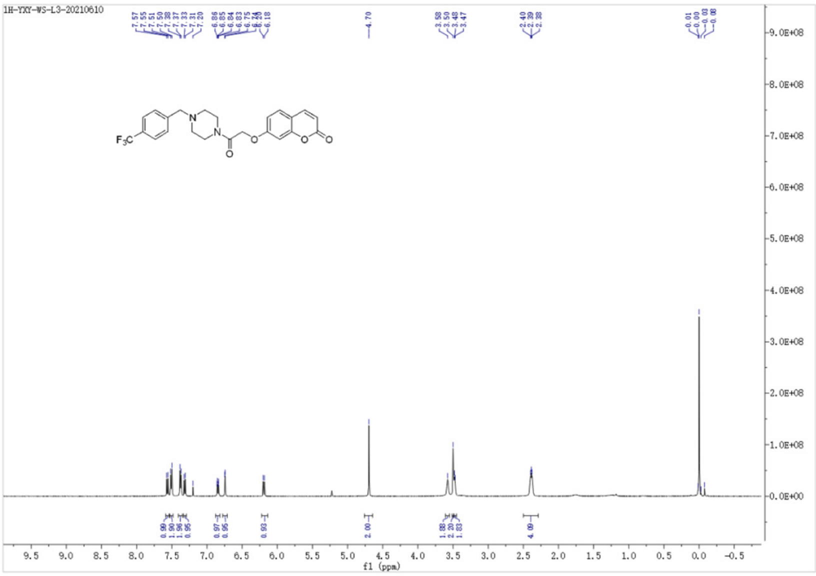

Figure S48. ^{1}H NMR spectrum **6o.**

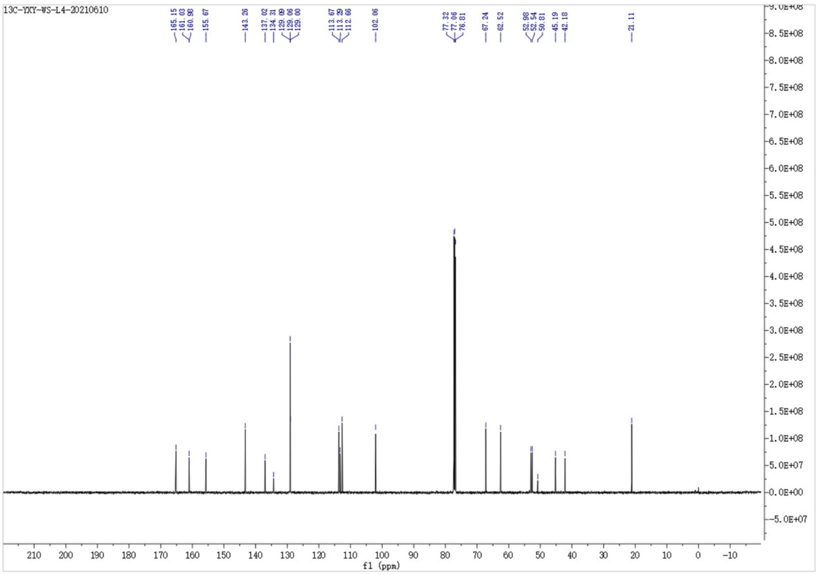

Figure S49. ^{13}C NMR spectrum **6o.**

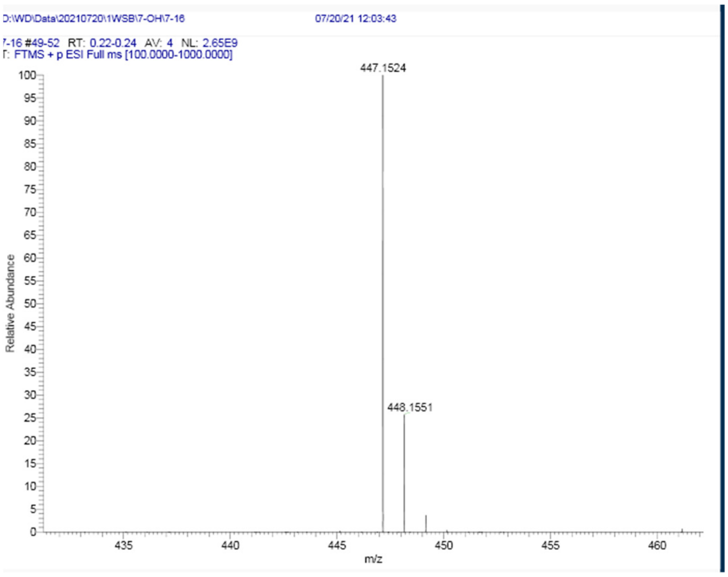

Figure S50. HRMS spectrum 6o.

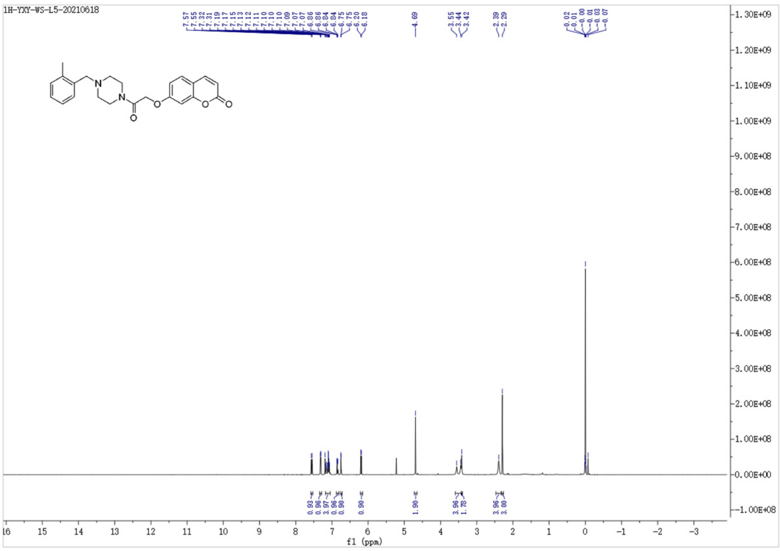

Figure S51. ^{1}H NMR spectrum **6p.**

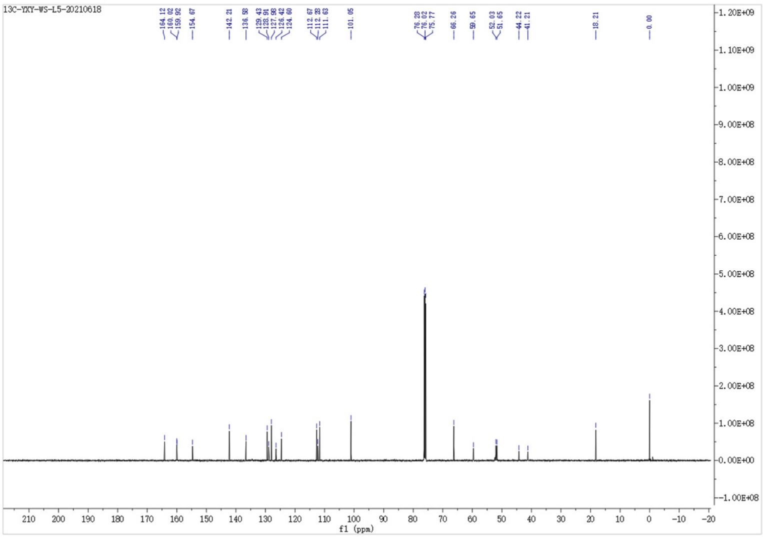

Figure S52. ^{13}C NMR spectrum **6p**.

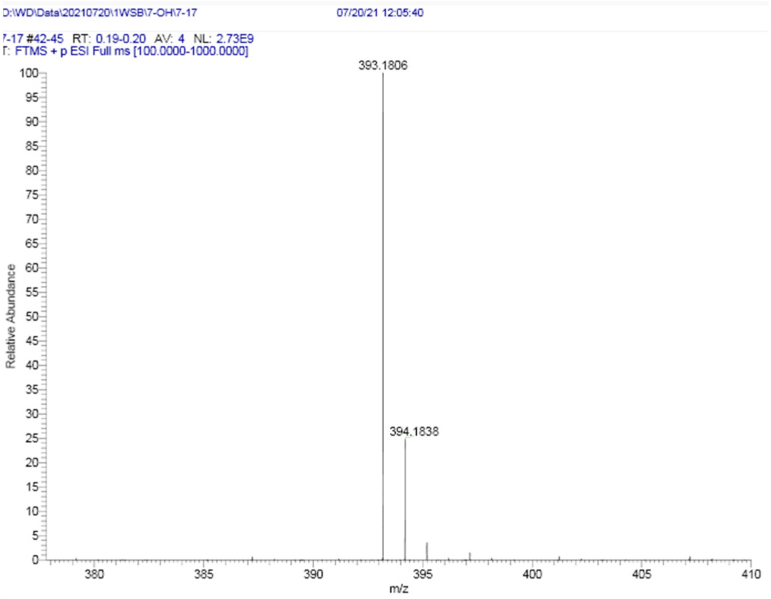

Figure S53. HRMS spectrum 6p.

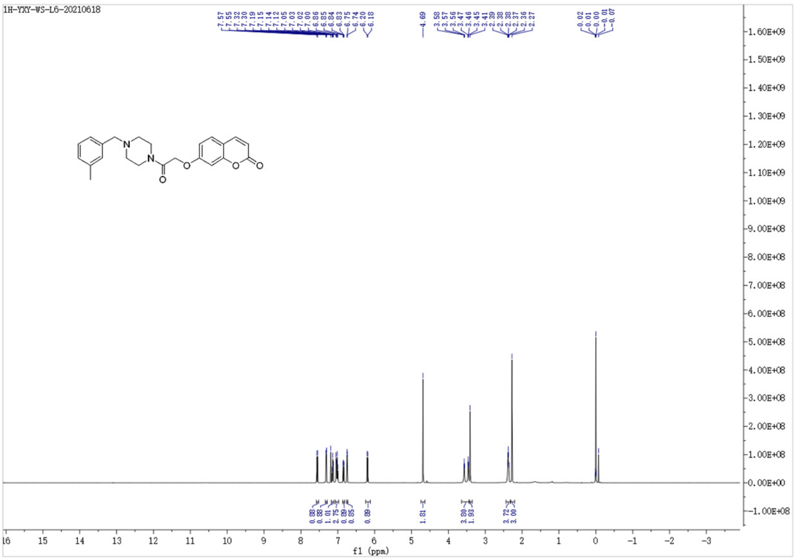

Figure S54. ^{1}H NMR spectrum **6q.**

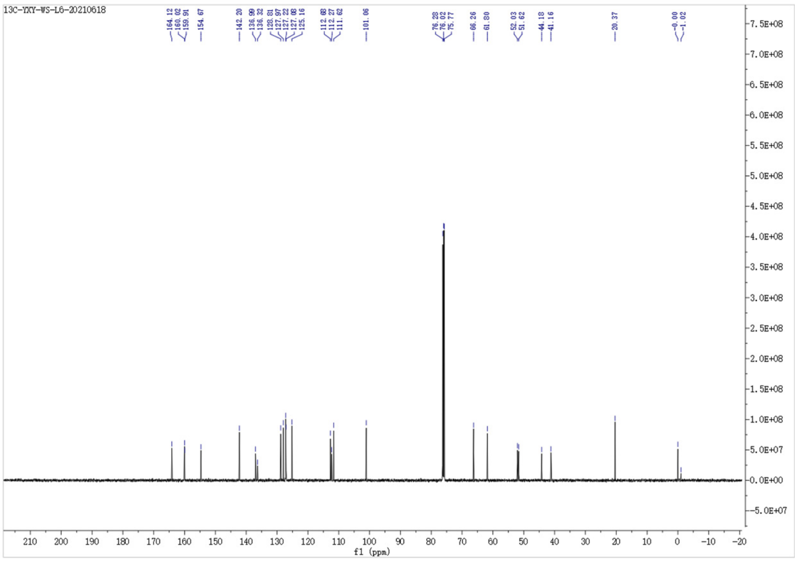

Figure S55. ^{13}C NMR spectrum **6q.**

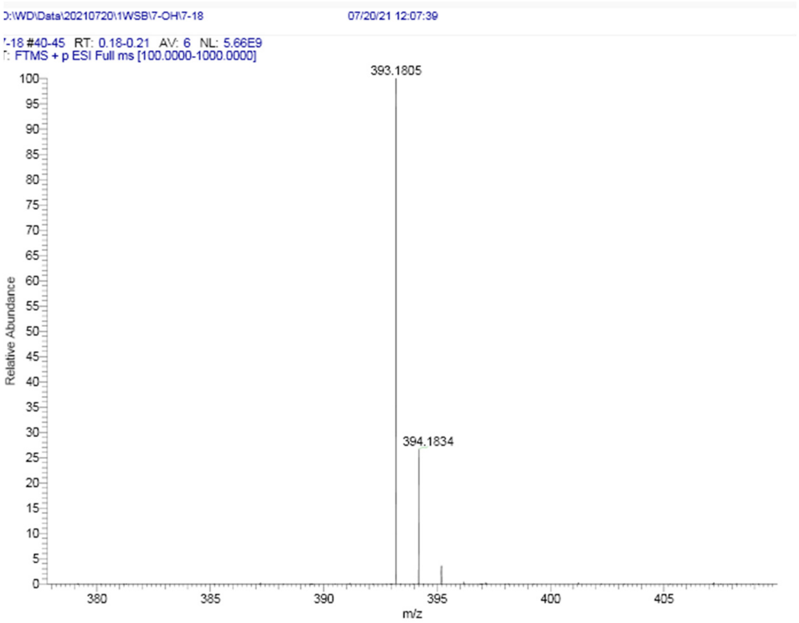

Figure S56. HRMS spectrum 6q.

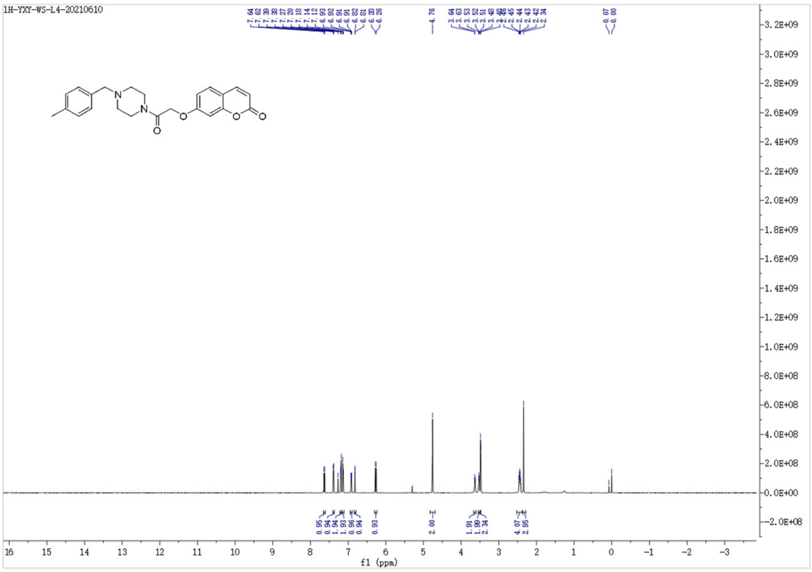

Figure S57. ^{1}H NMR spectrum **6r.**

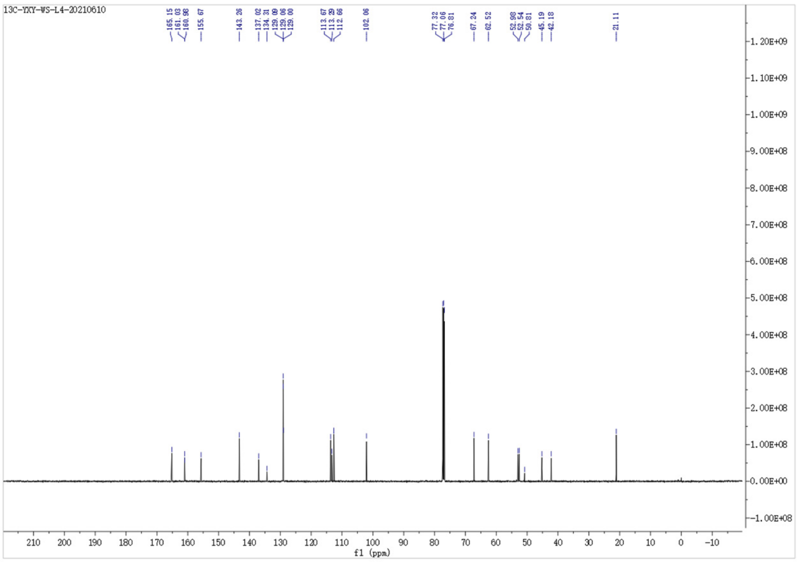

Figure S58. ^{13}C NMR spectrum **6r.**

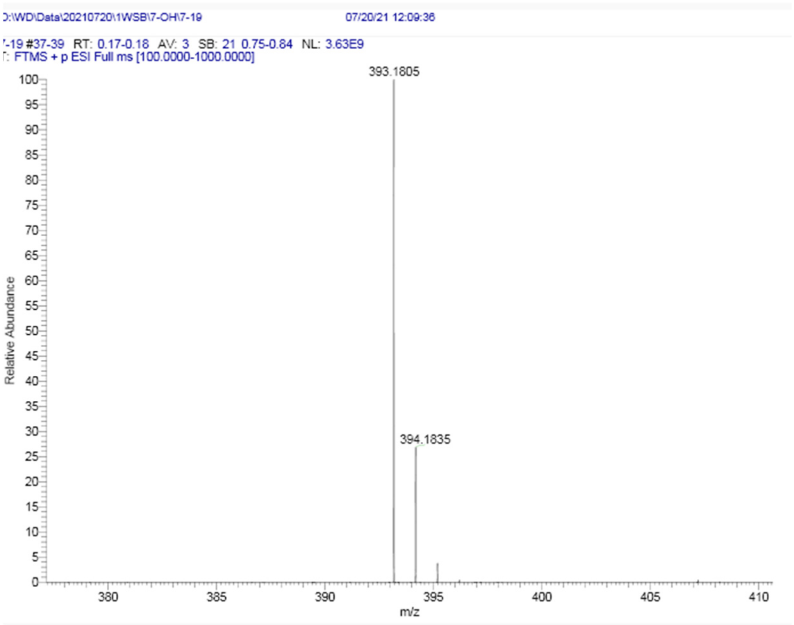

Figure S59. HRMS spectrum 6r.

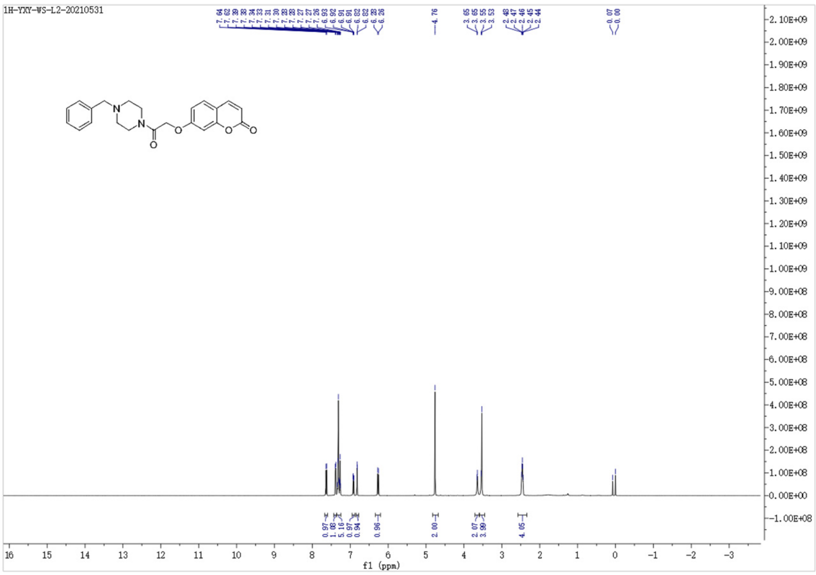

Figure S60. ^{1}H NMR spectrum **6s.**

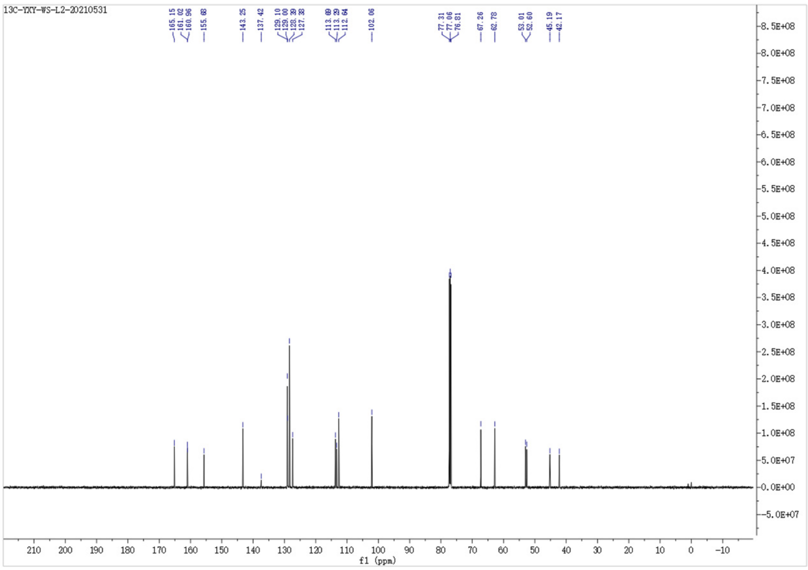

Figure S61. ^{13}C NMR spectrum **6s.**

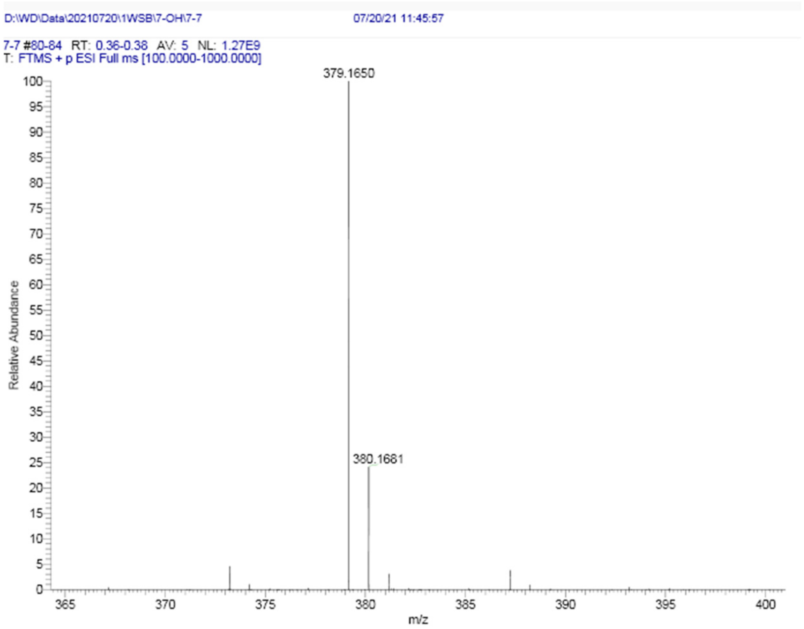

Figure S62. HRMS spectrum 6s.

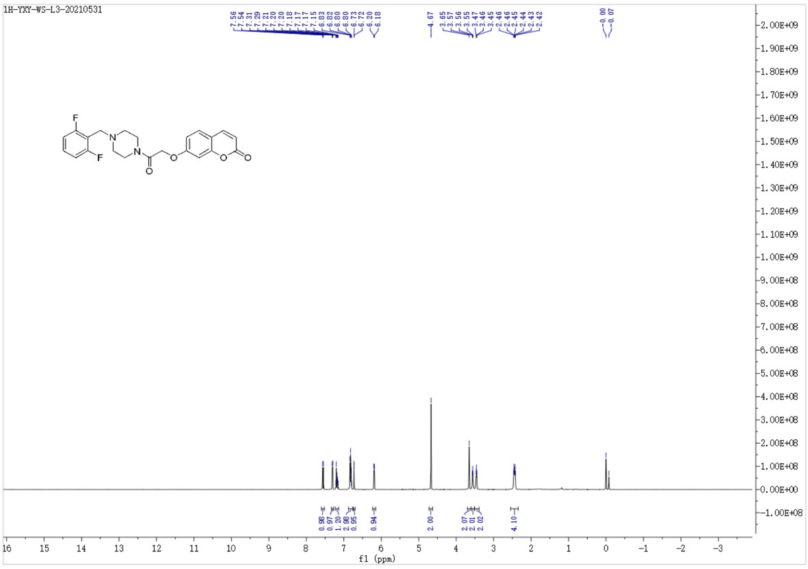

Figure S63. ^{1}H NMR spectrum **6t.**

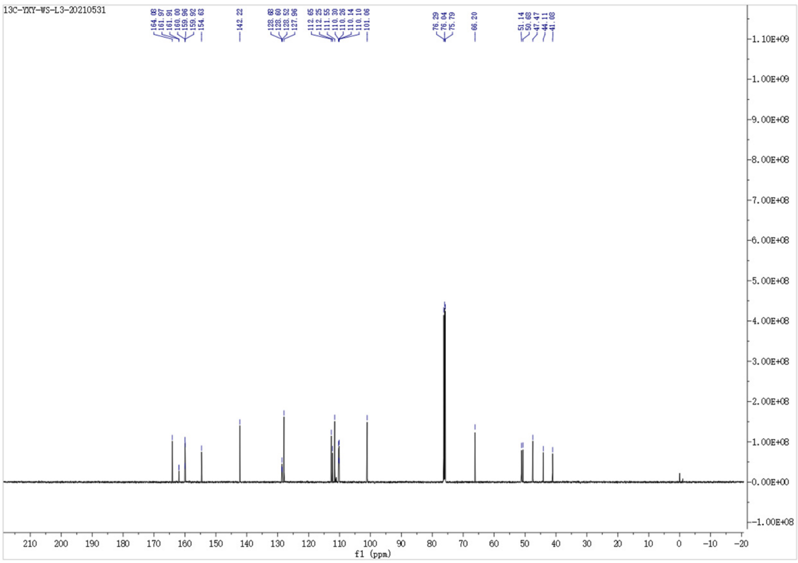

Figure S64. ^{13}C NMR spectrum **6t.**

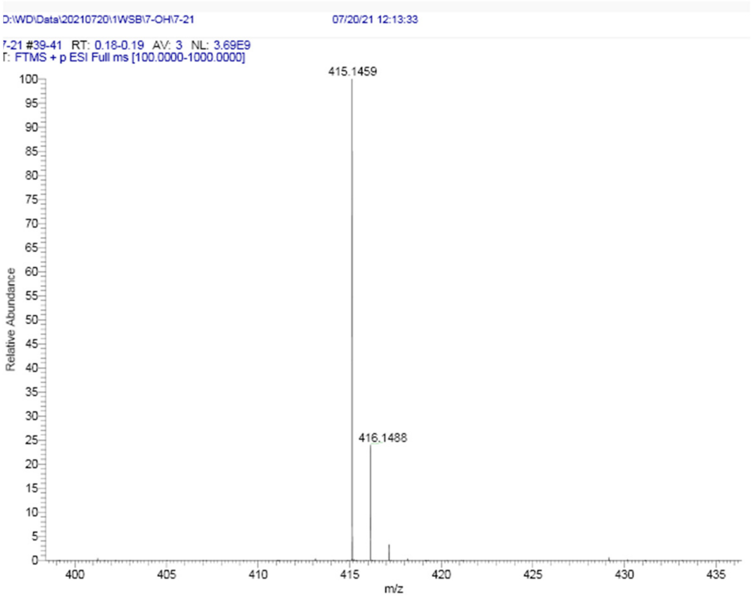

Figure S65. HRMS spectrum 6t.

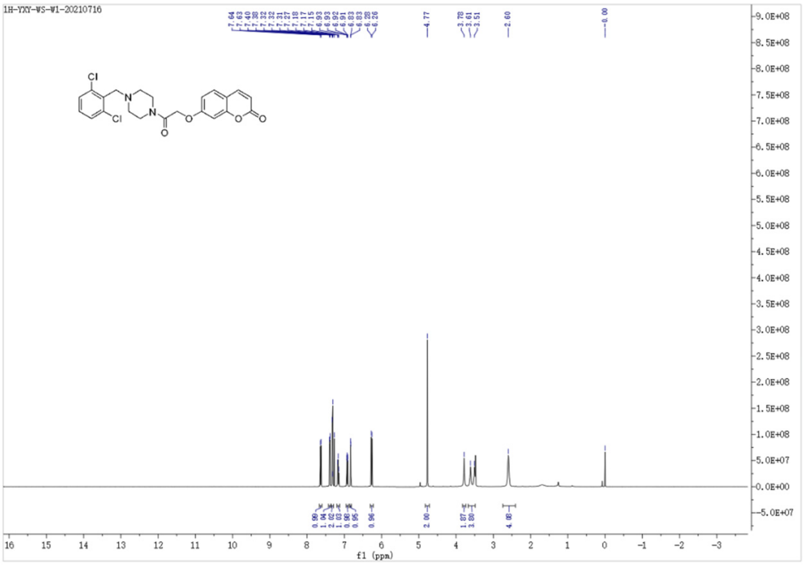

Figure S66. ^{1}H NMR spectrum **6u.**

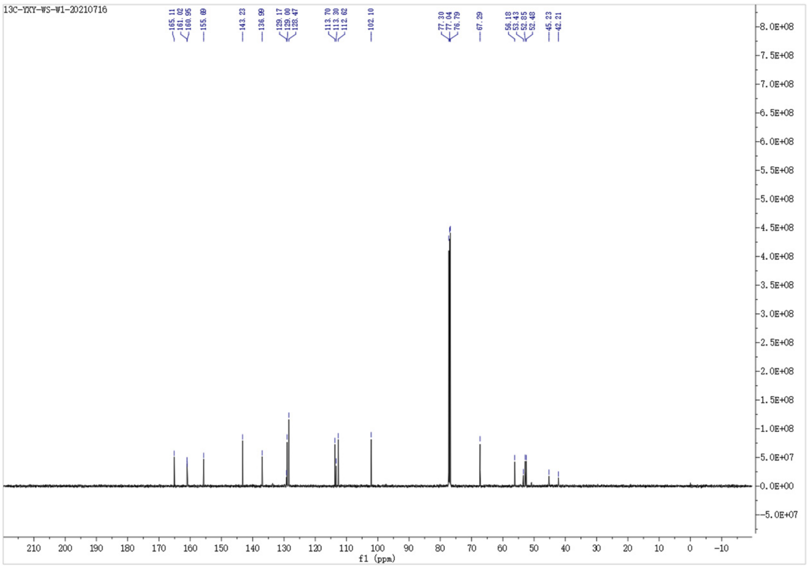

Figure S67. ^{13}C NMR spectrum **6u.**

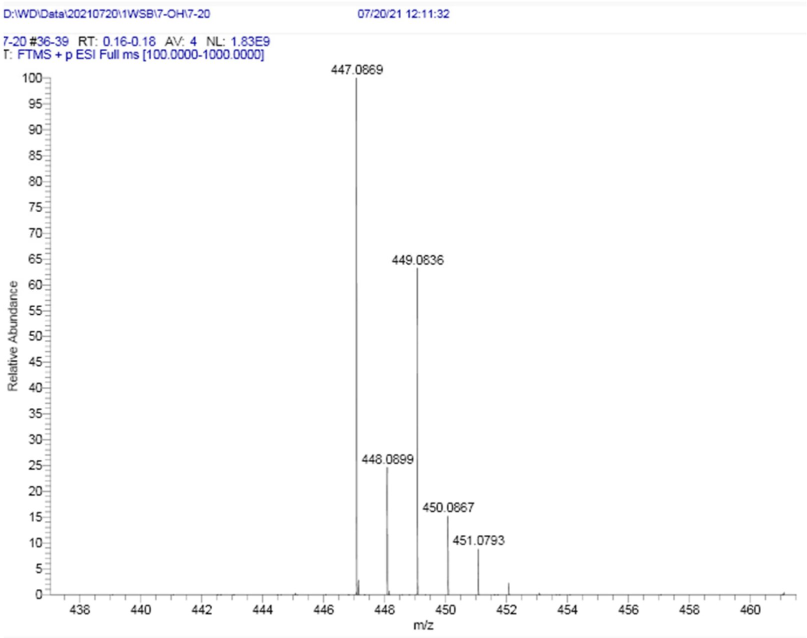

Figure S68. HRMS spectrum 6u.

References

1. Porsolt, R.D.; Bertin, A.; Jalfre, M. Behavioral despair in mice: A primary screening test for antidepressants. *Arch. Int. De Pharm. Et De.* **1977**, *229*, 327–336.
2. Zomkowski, A.D.; Santos, A.R.; Rodrigues, A.L. Evidence for the involvement of the opioid system in the agmatine antidepressant-like effect in the forced swimming test. *Neurosci. Lett.* **2005**, *381*, 279–283.
3. Zomkowski, A.D.E.; Rosa, A.O.; Lin, J.; Santos, A.R.S.; Calixto, J.B.; Rodrigues, A.L.S. Evidence for serotonin receptor subtypes involvement in agmatine antidepressant-like effect in the mouse forced swimming test. *Brain. Res.* **2004**, *1023*, 253–263. https://doi.org/10.1016/j.brainres.2004.07.041.
4. Steru, L.; Chermat, R.; Thierry, B.; Simon, P. The tail suspension test: A new method for screening antidepressants in mice. *Psychopharmacology* **1985**, *85*, 367–370, https://doi.org/10.1007/bf00428203.
5. Sairanen, M.; Lucas, G.; Ernfors, P.; Castrén, M.; Castrén, E. Brain-Derived Neurotrophic Factor and Antidepressant Drugs Have Different But Coordinated Effects on Neuronal Turnover, Proliferation, and Survival in the Adult Dentate Gyrus. *J. Neurosci.* **2005**, *25*, 1089–1094, https://doi.org/10.1523/jneurosci.3741-04.2005.
6. Elliott, P.J.; Chan, J.; Parker, Y.M.; Nemeroff, C.B. Behavioral effects of neurotensin in the open field: Structure-activity studies. *Brain Res.* **1986**, *381*, 259–265, https://doi.org/10.1016/0006-8993(86)90075-2.
7. Eckeli, A.L.; Dach, F.; Rodrigues, A.L.S. Acute treatments with GMP produce antidepressant-like effects in mice. *NeuroReport* **2000**, *11*, 1839–1843, https://doi.org/10.1097/00001756-200006260-00008.
